# A Rare Mutation in *SPLUNC1* Affects Bacterial Adherence and Invasion in Meningococcal Disease

**DOI:** 10.1093/cid/ciz600

**Published:** 2019-07-01

**Authors:** Bayarchimeg Mashbat, Evangelos Bellos, Stephanie Hodeib, Fadil Bidmos, Ryan S Thwaites, Yaxuan Lu, Victoria J Wright, Jethro A Herberg, Daniela S Klobassa, William G Walton, Werner Zenz, Trevor T Hansel, Simon Nadel, Paul R Langford, Luregn J Schlapbach, Ming-Shi Li, Matthew R Redinbo, Y Peter Di, Michael Levin, Vanessa Sancho-Shimizu

**Affiliations:** 1 Department of Paediatric Infectious Diseases, Division of Medicine, Imperial College London, Norfolk Place, United Kingdom; 2 National Heart and Lung Institute, Imperial College London, United Kingdom; 3 Department of Pediatric and Adolescence Surgery, Division of General Pediatric Surgery, Medical University Graz, Austria; 4 Paediatric Intensive Care Unit, St. Mary’s Hospital, Imperial College Healthcare Trust, London, United Kingdom; 5 Faculty of Medicine Brisbane, The University of Queensland Brisbane, Australia; 6 Paediatric Critical Care Research Group, The University of Queensland Brisbane, Australia; 7 Paediatric Intensive Care Unit, Lady Cilento Children’s Hospital, Children’s Health Queensland, Brisbane, Australia; 8 Department of Pediatrics, Bern University Hospital, Inselspital, University of Bern, Switzerland; 9 Department of Chemistry, University of North Carolina, Chapel Hill; 10 Department of Biochemistry and Biophysics, University of North Carolina, Chapel Hill; 11 Lineberger Comprehensive Cancer Center, University of North Carolina, Chapel Hill; 12 Department of Environmental and Occupational Health, University of Pittsburgh, Pennsylvania

**Keywords:** severe infectious disease, meningococcal disease, mucosal immunity, human genetics, sepsis

## Abstract

**Background:**

*Neisseria meningitidis* (Nm) is a nasopharyngeal commensal carried by healthy individuals. However, invasive infections occurs in a minority of individuals, with devastating consequences. There is evidence that common polymorphisms are associated with invasive meningococcal disease (IMD), but the contributions of rare variants other than those in the complement system have not been determined.

**Methods:**

We identified familial cases of IMD in the UK meningococcal disease study and the European Union Life-Threatening Infectious Disease Study. Candidate genetic variants were identified by whole-exome sequencing of 2 patients with familial IMD. Candidate variants were further validated by in vitro assays.

**Results:**

Exomes of 2 siblings with IMD identified a novel heterozygous missense mutation in *BPIFA1*/*SPLUNC1*. Sequencing of 186 other nonfamilial cases identified another unrelated IMD patient with the same mutation. SPLUNC1 is an innate immune defense protein expressed in the nasopharyngeal epithelia; however, its role in invasive infections is unknown. In vitro assays demonstrated that recombinant SPLUNC1 protein inhibits biofilm formation by Nm, and impedes Nm adhesion and invasion of human airway cells. The dominant negative mutant recombinant SPLUNC1 (p.G22E) showed reduced antibiofilm activity, increased meningococcal adhesion, and increased invasion of cells, compared with wild-type SPLUNC1.

**Conclusions:**

A mutation in *SPLUNC1* affecting mucosal attachment, biofilm formation, and invasion of mucosal epithelial cells is a new genetic cause of meningococcal disease.

## Teaser

Severe meningococcal disease may result from single-gene inborn errors of immunity, affecting bacterial colonization, biofilm formation, and invasion. Our findings demonstrate the host protective role SPLUNC1 plays in maintaining mucosal immunity in the upper respiratory tract, and preventing invasive disease.

*Neisseria meningitidis* (Nm) colonizes the epithelia of the nasopharynx in the upper respiratory tract in a high proportion of healthy children and adults intermittently throughout life. In Europe and the United States, carriage rates range from 10–30% [1], although the majority of individuals colonized by the bacteria do not develop invasive disease and remain asymptomatic. In less than 1% of carriers, the bacteria infiltrate the epithelial barrier and enter the bloodstream, causing life-threatening sepsis and/or meningitis [2], often with devastating consequences. The incidence rates of invasive meningococcal disease (IMD) in the prevaccine era were 5–10 cases per 100 000 children per year in Europe and North America [3]. Although antibiotic treatment is highly effective, the mortality rate remains between 8–14% [4]. In addition, a significant proportion of survivors have long-term sequelae, including hearing loss, other neurological deficits, skin scarring, or amputation [5].

The critical role of bactericidal antibodies in protection against IMD is well documented [6, 7]. Furthermore, innate immune effectors—in particular, in the complement system—are also essential in controlling Nm replication. Pathogen recognition receptors, such as toll-like receptor (TLR)4 and TLR2, which bind Nm lipopolysaccharide (LPS) and porin B, activate NF-kB, leading to pro-inflammatory cytokine production [8]. Epithelial cells in the upper airway produce several antimicrobial peptides and surfactant proteins to combat Nm infection, including cathelicidin LL-37 and surfactant proteins -A and -D [9, 10].

Genetic variants affecting components of the host immune response have been shown to underlie IMD [11]. Increased sibling risk ratios, λ _s,_ in children with IMD have been reported, implicating host genetics in disease susceptibility [12]. A large multi-country genome wide association study complement factor H established that polymorphisms in the *CFH* region were associated with susceptibility to IMD [13, 14]. Candidate gene studies have implicated a number of other genes in either susceptibility to or severity of disease, including the interleukin 1 gene cluster, comprising *IL1RN* and *IL1β* [15, 16], and an insertion deletion in the coagulation gene *SERPINE1* [17]. Monogenic disorders of the central complement element C3, the alternative pathway (factor D, properdin, factor H, and factor I), and terminal components of the complement system (C5–C9) have been well documented to confer selective susceptiblity to IMD [18–22]. Additionally, patients with mutations in genes involved in signal transduction downstream of TLRs, autosomal recessive (AR) interleukin-1 receptor-associated kinase 4 (*IRAK4*) [23], and AR NF-kappa-B essential modulator (*NEMO*) deficiencies [24], usually linked to invasive pneumococcal infections, have been described as suffering from IMD. Rare variants in *F5* and *TLR4* have also been implicated in severity and susceptibility to disease [25, 26].

Although the studies described above provide evidence of a strong genetic contribution to IMD susceptibility, the genes identified to date explain only a small proportion of IMD patients. In an effort to uncover new genetic aetiologies of IMD, we employed whole-exome sequencing (WES) of well-defined familial cases that led to the identification of a novel mutation in *SPLUNC1*. This gene encodes for the secreted BPIFA1/SPLUNC1 (25 kDa) protein, expressed in the upper respiratory tract. SPLUNC1 has been studied in the context of respiratory infections and inferred to prevent infections by inhibiting bacterial biofilm formation [27–31]. Given that this secreted protein with surfactant properties is highly expressed in the nasopharyngeal epithelial tissues [32, 33], a site of Nm colonization, and that polymorphisms in *SPLUNC1* have been associated with impaired airway functions [34–36], we hypothesized that the implicated genetic modification may confer increased susceptibility to IMD.

## MATERIALS AND METHODS

### Study Cohorts

The UK meningococcal disease cohort included participants diagnosed with meningococcal disease and enrolled through several recruitment sites, including St Mary’s Hospital London, the Meningitis Research Foundation, and Alder Hey Children’s Hospital in Liverpool. Complete clinical details of the participants have been previously described elsewhere [13].

The European Union Childhood Life-Threatening Infectious Disease Study (EUCLIDS) included febrile children presenting to hospital with a suspected bacterial infection [37]. A total of 549 patients with invasive bacterial infections were selected for WES, of which 220 were diagnosed with meningococcal disease. Meningococcal disease was diagnosed in persons presenting with petechial or purpuric rash and meningitis and/or septicaemia. Confirmation of the disease was made by a bacterial culture or polymerase chain reaction from blood or cerebrospinal fluid.

Nasal fluid samples were collected from healthy infants under ethical approval from the Black Country Research Ethics committee (reference 15/WM/0343).

### Nasal Fluid Collection

Nasal fluids were collected using Nasosorption FXi-13 devices (Mucosal Diagnostics UK, Ltd.), which were manipulated into the nostril lumen and depressed for 30 seconds. Samples were then eluted from Nasosorption devices in phosphate-buffered saline (PBS) with 1% BSA and 0.05% Tween-20. Subsequently, SPLUNC1 expression was detected in these samples using standard Western blotting with a goat antihuman SPLUNC1 primary antibody (R&D Systems, Cat# AF1897) and rabbit antigoat horseradish peroxidase-conjugated detection (Cat# SC2768) antibody. As a method to control for sample loading, human serum albumin expression was determined simultaneously with the use of a DuoSet enzyme-linked immunosorbent assay (ELISA) kit (R&D Systems Cat# DY1455), following the manufacturer’s recommendation.

### Whole-exome Sequencing and Variant Annotation

The patients’ genomic DNA samples were extracted using a standard laboratory technique and subjected to genomic DNA library preparation and capture at Oxford Gene Technology (Oxfordshire, United Kingdom). Protein-coding regions were targeted using Agilent’s SureSelect Human All Exon v4 platform. The enriched libraries were sequenced on Illumina HiSeq 2000, achieving an average target coverage of ~44 x across samples, with 85% of exonic bases sequenced at least 10x. The resulting paired-end 100 base pair–long sequencing reads were aligned to the human genome (assembly version GRCh37) using bwa v0.7 [38]. De-duplication and sequencing quality assessment were performed using the Picard tools (http://broadinstitute.github.io/picard), followed by joint sample genotyping with genome analysis tool kit [39]. The resulting single nucleotide polymorphism (SNP) and indel variant calls were functionally annotated using Ensembl’s Variant Effect Predictor [40]. Finally, to leverage familial relationships in our samples, we developed an algorithm that prioritized variants that appeared to be identical by descent in the individuals of interest.

### Expression and Purification of Human SPLUNC1

We performed recombinant SPLUNC1 (rSPLUNC1) protein mutagenesis, expression, and purification as previously described [41, 42]. Briefly, the G22E and N-terminal deleted (ΔN) mutant constructs were created from the full-length SPLUNC1 cDNA using polymerase chain reaction mutagenesis. Wild-type (WT) and mutated SPLUNC1 cDNAs were cloned into pMCSG7 for protein expression. BL21-CodonPlus competent cells were transformed with the expression plasmid and grown in Luria-Bertani (LB) broth supplemented with an antibiotic cocktail to reach an optical density (OD_600_) of 0.6. Expression was induced by adding 0.1 mM of isopropyl-1-thio-D-galactopyranoside. The soluble fraction was purified using an Ni-NTA His-trap gravity column and analyzed with a S200 gel filtration column on an ÄKTAxpress (GE Healthcare).

### *Neisseria meningitidis* Growth Assay in the Presence of Recombinant SPLUNC1

The bactericidal activity of SPLUNC1 was tested by incubating the WT MC58 Nm strain with varying concentrations of human rSPLUNC1. Nm were grown on brain heart infusion (BHI) agar at 37°C in 5% CO_2_ overnight. The next day, bacteria were subcultured in BHI broth to reach mid-log phage growth and the culture was adjusted to an OD_600_ of 0.1 (10^8^ colony forming unit [CFU] per mL). For individual experiments, approximately 10^6^ cells were incubated alone or in the presence of increasing amounts of rSPLUNC1 (1 µg, 5 µg, and 10 µg) or the control protein, BSA. At time points from 0–24 hours, aliquots were collected and plated out on BHI agar to determine the viable counts of surviving bacteria.

### *Neisseria meningitidis* Adhesion and Invasion Assays

Human bronchial epithelial cell line (16HBE14) cells were grown in Dulbecco’s Modified Eagle Medium (DMEM), supplemented with 10% fetal bovine serum at 37°C in 5% CO_2_. Nm cells grown on BHI agar plates were suspended in PBS to achieve an multiplicity of infection of 1 for both adherence and invasion assays, as previously described [43]. Briefly, the confluent monolayer of epithelial cells was infected with Nm in the presence or absence of rSPLUNC1 or BSA (10 µg per ml). For the adherence assay, after 4 hours, wells were washed 3 times with PBS and incubated with 1% saponin for 10 minutes at 37°C. For the invasion assay, gentamicin (Sigma) was added to each well at a final concentration of 150 µg/ml and incubated for 1 hour at 37°C. Wells were washed 3 times with PBS and subsequently incubated with saponin as above, and viable counts were determined by CFU counting. Data are presented as the percentage of CFUs, compared to infecting inoculum. Each condition tested was carried out in triplicate at least 3 independent times.

### Lipopolysaccharide Binding Assay

A slightly modified version of the ELISA-based LPS binding test was used to measure the LPS binding abilities of SPLUNC1 variants. Briefly, a 96-well plate was coated with purified LPS (250 ng) from Nm serogroup B (courtesy of Peter Van Der Ley, Institute for Translational Vaccinology, Intravacc) or *Salmonella* Minnesota R595 (InvivoGen) overnight. The rest of the steps were performed as per protocol [44]. Absorbance readings were analyzed using SoftMax Pro software (Molecular Devices). The results are based on at least 3 independent experiments and represented as means ± standard errors of the mean.

### Biofilm Biomass Assay

A modified version of a previously described microtiter assay was used to assess the meningococcal surface attachment to an abiotic surface. Briefly, WT and pili deficient ( *pilE*-) MC58 strains were suspended in DMEM to obtain the OD_600_ of 0.5 from the overnight growth from supplemented BHI plates. Assay wells were incubated with SPLUNC1 protein or BSA for 3 hours at 37°C. Wells containing bacteria inoculated without the addition of recombinant protein were also used as controls. Biofilm-forming microcolonies were stained for 15 minutes using 0.5% aqueous crystal violet; were washed twice in water to remove excess stain; had 80% ethanol added; and had ODs measured at 590 nm.

### Statistical Analysis

We assessed differences between controls and SPLUNC1 proteins in bacterial growth, biofilm, and adhesion or invasion assays using 1- or 2-way analyses of variance, followed by a Tukey’s multiple comparison test or Student’s *t*-test. Unless otherwise stated, a *P* < .05 was considered statistically significant for all analyses.

## RESULTS

### Whole-exome Sequencing of Familial Invasive Meningococcal Disease Cases

Children with confirmed IMD, sepsis, and/or meningitis were recruited through the UK meningococcal disease study [13] and the EUCLIDS [37]. We performed WES on multiplex families with confirmed diagnoses of IMD. We focused on a family (kindred A) with 2 siblings who suffered from meningococcal sepsis, with incidences occurring 5 years apart (see Supplementary Table 1). They were both found to have normal, functional, complement-mediated killing of Nm [45] and specific bactericidal antibodies. Clinical investigations excluded complement or antibody deficiencies (see Supplementary Figure 1). Due to its breadth of coverage, WES identified multiple candidate variants that appeared to have been shared by the siblings. However, many of these variants needed to be excluded, as they were inherited from a different parent in each of the siblings and were, thus, only identical by state and not by descent [46, 47]. Since the parent DNA was not available, we could not perform pedigree-based phasing to directly determine identity by descent (IBD) in our siblings. Instead, we undertook population-based phasing using SHAPEIT [48], and then cross-referenced the resulting haplotype blocks to obtain IBD genomic segments. This allowed us to adopt a modified familial filtering approach that began by excluding variants falling outside IBD blocks. Within the shared segments, we limited our search to novel or rare sequence variants (<1% minor allele frequency). Variants were annotated using Ensembl’s Variant Effect Predictor [40], excluding synonymous variants and those that were predicted to have a low impact on the resulting protein. Using this filtration, we identified 231 variants in the siblings (see Supplementary Table 2). To narrow down the candidate variants, Combined Annotation Dependent Depletion (https://cadd.gs.washington.edu/) and bidirectional Sorting Intolerant from Tolerant [49] scores were calculated, highlighting those variants that were likely to impact on function. The prioritizing of genes, using the gene damage index, was also incorporated into the analyses. No mutations consistent with any of the known genetic aetiologies underlying the known primary immunodeficiencies were identified [50] (see Supplementary Table 2). We prioritized *SPLUNC1—*also known as *BPIFA1,* and which harbored a novel mutation—as a candidate, due to its known role in host defense against Gram-negative bacteria [27, 44].

**Figure 1.**
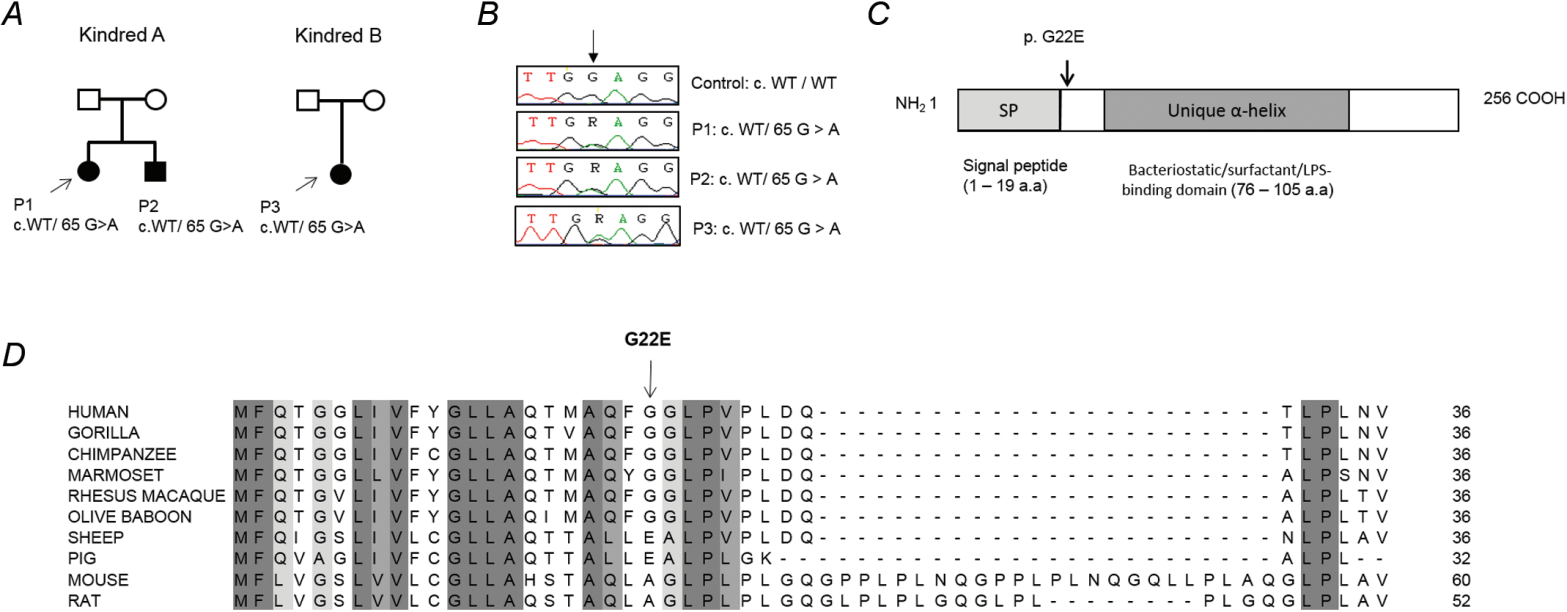
A heterozygous missense *BPIFA1/SPLUNC1* mutation identified in invasive meningococcal disease (IMD) patients. A novel missense c.65 G > A, p.G22E *SPLUNC1* mutation was found in 3 IMD patients in 2 kindreds. *A,* Pedigrees of the 2 families’ affected individuals are shaded in black and probands are indicated by arrows. *B,SPLUNC1* sequencing from control and patients. Arrow indicates the position of the mutation. *C,* Schematic representation of the SPLUNC1 protein, with the signal peptide domain (SP) and BPI–alpha/beta homologous domain (BPI-α/β) indicated.) *D,* Amino acid sequence alignment showing conservation of the N-terminal domain of SPLUNC1 across different species. Abbreviation: COOH, C-terminal domain of proteins; IMD, invasive meningococcal disease LPS, lipopolysaccharide; WT, wild-type.

SPLUNC1 is a secreted protein expressed exclusively in the nasopharyngeal tract, with proportionately higher expression in the nasal lining fluid of children compared with adults (Supplemental Figure 4*B*).The novel *SPLUNC1* missense mutation changes a guanine to an alanine residue at nucleotide position 65, leading to glutamic acid in place of a glycine at amino acid position 22 (Figure 1A). Further targeted sequencing of 186 IMD patients from the UK meningococcal disease study [13] revealed an unrelated case (P3; kindred B) who also carried the same heterozygous *SPLUNC1* mutation (Figure 1A and 1B; see Supplementary Table 2). Sanger sequencing in all 3 patients subsequently confirmed the variant. WES of P3 revealed no variants corresponding to any known primary immunodeficiencies. This *SPLUNC1* (c.65G > A, p.G22E) variant was absent from known public databases (dbSNP, EVS, gnomAD, and Bravo 1k Genomes) and from our in-house database, which comprises 549 exomes of children with life-threatening infections (Figure 1B). The p.G22E mutation affects the N-terminus of the SPLUNC1 protein (Figure 1C), which is unique from the other bactericidal permeability increasing family protein sequences and has been shown to regulate epithelial sodium transport, influencing mucociliary clearance in the host airway [41]. Moreover, the glycine residue is well conserved across primates and differs from other species (Figure 1D), reflective of the diversity in function and pH dependency of the N-terminal domain of the protein along the evolutionary tree [41].

### Recombinant SPLUNC1 Inhibits *Neisseria meningitidis* Biofilm Formation

**Figure 2.**
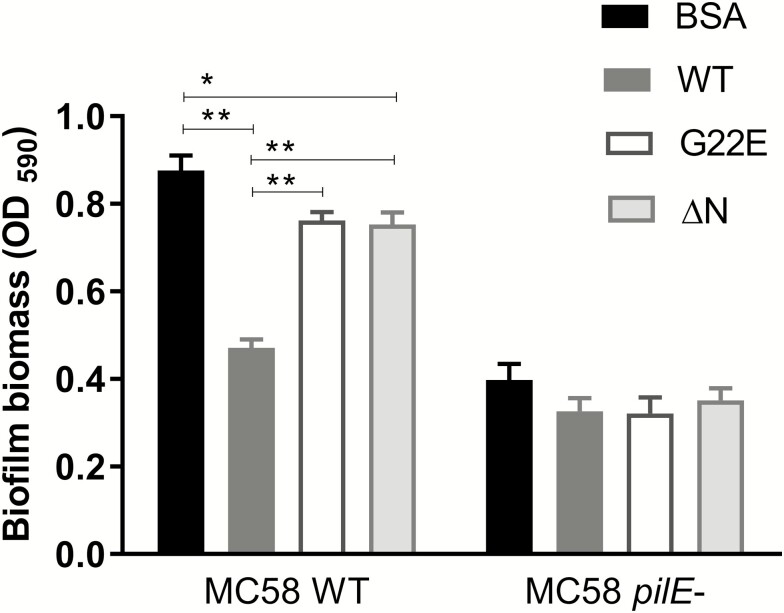
Nm biofilm formation is inhibited by rSPLUNC1. Biofilm biomass formed by WT or *pilE*-mutant Nm in media supplemented with BSA, WT, mutant G22E, or ΔN mutant rSPLUNC1 proteins are shown on a microtitre plate. Results are expressed as the means ± SEMs from 3 independent experiments. Statistical significance was assessed using a 2-way ANOVA, followed by Tukey’s test. *P < .05; **P < .001. Abbreviations: ΔN, N-terminal deleted; ANOVA, analysis of variance; Nm, *Neisseria meningitidis*; OD, optical density; *pilE*, pili deficient; rSPLUNC1, recombinant SPLUNC1; SEM, standard error of the mean; WT, wild-type.

The rSPLUNC1 protein was previously shown to exert surface tension–reducing properties in airway secretions, consistent with its surfactant-like properties, which are responsible for the inhibition of biofilm formation by various pathogens in the airway [27, 29, 51, 52]. We assessed this surfactant property of SPLUNC1 in the context of Nm biofilm formation using a standard microtitre plate–based bacterial biofilm assay [53]. Biofilm formation was measured on the surface of a microtitre plate in the presence of BSA, WT, or mutant G22E rSPLUNC1 proteins (Figure 2). The SPLUNC1 protein constructs were generated using mutagenesis, and protein expression and purification were performed using previously described methods [42]. The Nm MC58 *pilE-* strain was included as a control, as it is impaired in its ability to form biofilm, compared with its isogenic parent Nm MC58 WT [54]. Since the G22E mutation is located in the N-terminus, we also used a mutant protein containing a deletion of its entire N-terminal domain (ΔN rSPLUNC1; amino acids 1–44) [41]. The presence of the WT rSPLUNC1 protein significantly reduced (*P* < .0001) the biofilm formation of Nm, compared with BSA (Figure 2; see Supplementary Figure 2). Both mutant p.G22E and ΔN rSPLUNC1 proteins elicited an impaired capacity to inhibit biofilm formation of Nm. No significant difference in biofilm formation was observed with any of the proteins by the isogenic *pilE-* mutant, which lacks the natural ability to form biofilm on a polystyrene surface [54].

### Recombinant SPLUNC1 has no Bactericidal Effect on *Neisseria meningitidis* Growth

**Figure 3.**
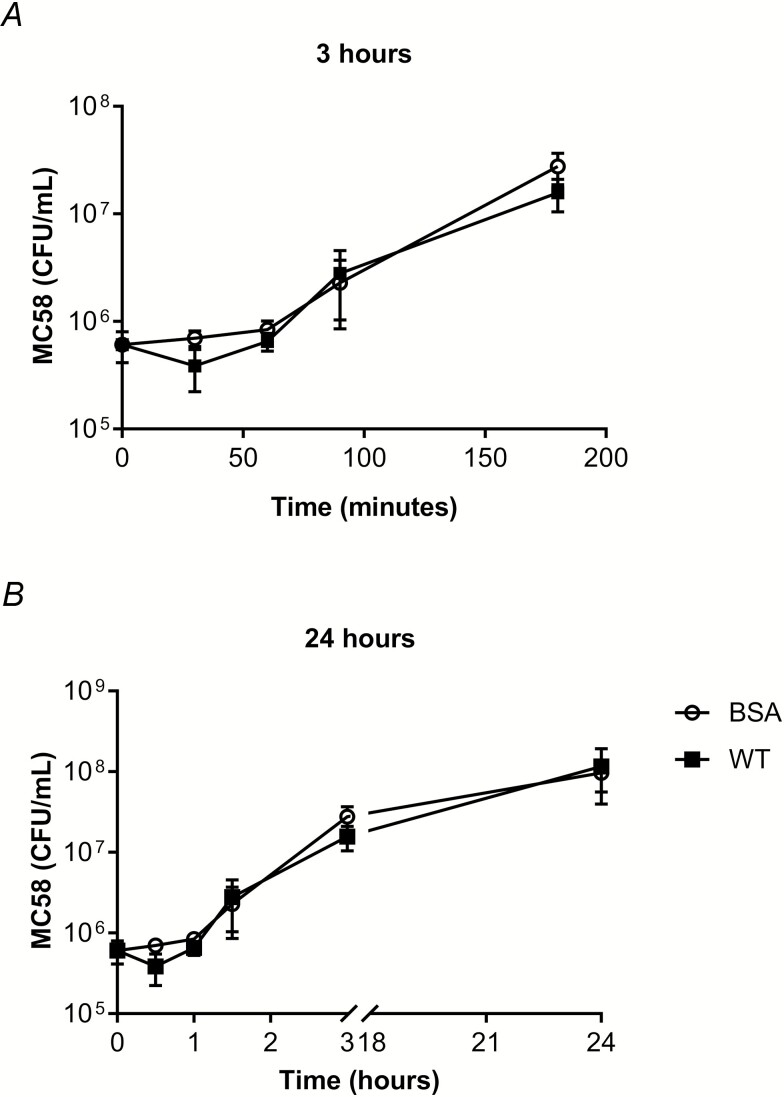
Nm growth is not affected by rSPLUNC1. Nm was grown in the presence of WT rSPLUNC1 or BSA for up to (*A*) 3 hours or (*B*) 24 hours. The bacterial viability was assessed by counting CFUs at regular intervals during infection. Results are expressed as means ± SDs from at least 3 independent experiments carried out in triplicates. Statistical significance was assessed using the Student’s *t*-test. Abbreviations: Nm, *Neisseria meningitidis*; rSPLUNC1, recombinant SPLUNC1; SD, standard deviation; WT, wild-type.

SPLUNC1 has been reported to elicit bacteriostatic properties against a number of Gram-negative bacteria, including *Pseudomonas aeruginosa* [30] and *Burkholderia cenocepacia* [42]. In order to investigate whether rSPLUNC1 has any direct bactericidal effect on Nm growth, a bacterial viability assay was performed in the presence of WT rSPLUNC1. Our results showed that rSPLUNC1 did not elicit bactericidal activity on Nm growth under the conditions tested (Figure 3A; see Supplementary Figure 3). We did not detect a difference in bacterial growth in the presence of rSPLUNC1 or BSA during the 3-hour incubation period. Similar results were found when bacterial viability was assessed over 24 hours (Figure 3B).

### Recombinant SPLUNC1 Inhibits *Neisseria meningitidis* Adhesion and Invasion

**Figure 4.**
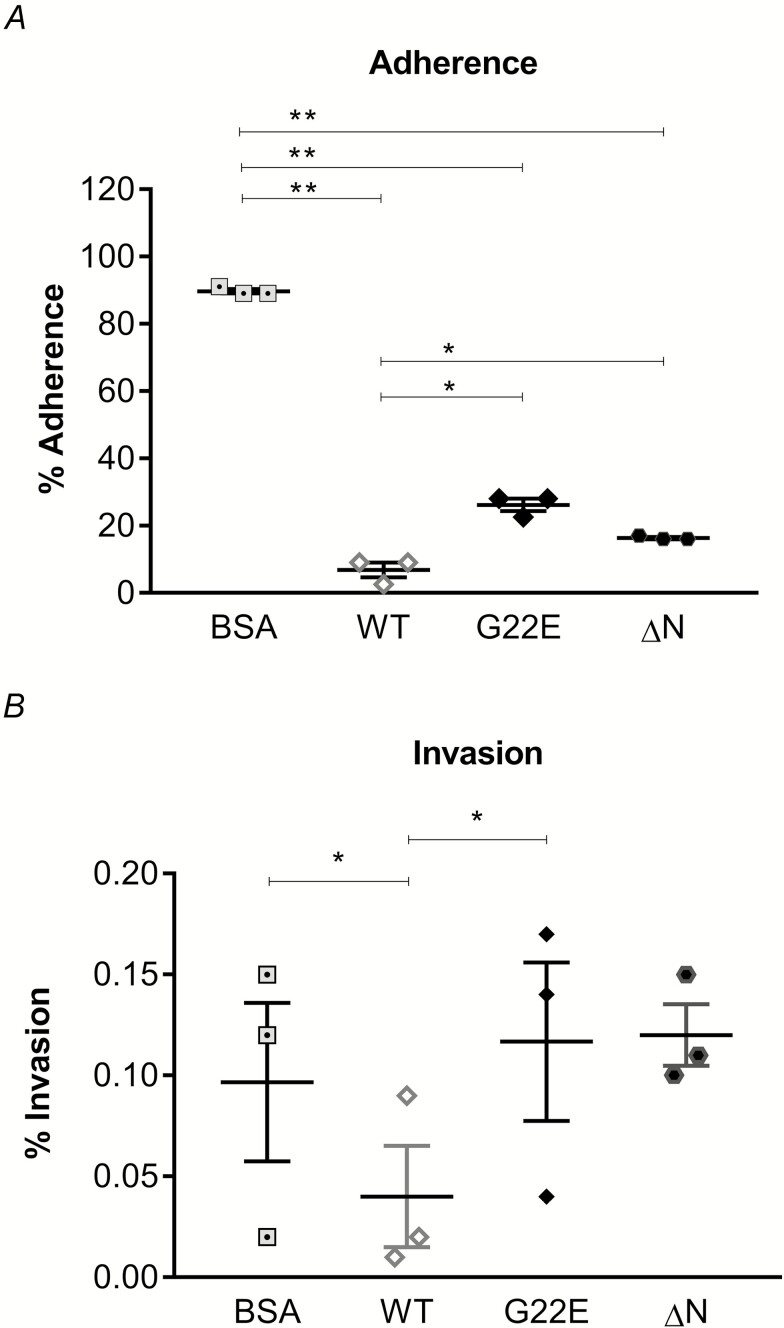
Nm adherence and invasion of 16HBE14 is inhibited by rSPLUNC1. The effects of BSA, WT, mutant G22E, or ΔN mutant rSPLUNC1 proteins on (*A*) Nm adherence or (*B*) invasion into 16HBE14 cells were assessed. Each condition was carried out in triplicate and the means ± SEMs from 3 independent experiments, following 4 hours incubation, are shown. *P < .05, **P < .001: determined by 1-way ANOVA with Tukey’s test. Abbreviations: ΔN, N-terminal deleted; 16HBE14, human bronchial epithelial cell line; ANOVA, analysis of variance; Nm, *Neisseria meningitidis*; rSPLUNC1, recombinant SPLUNC1; SEM, standard error of the mean; WT, wild-type.

The nasopharyngeal acquisition of Nm via close contact with saliva or respiratory droplets can lead to prolonged colonization of the nasopharynx or lead to invasive disease [1]. Patient cells were unavailable, so a human airway cell line was used to carry out molecular investigations of this protein. To assess whether SPLUNC1 is involved in early events of invasive infection by Nm, we compared adherence and invasion of Nm in 16HBE14 in the presence of WT or mutant G22E rSPLUNC1 proteins. An immunoblotting analysis from whole-cell lysates confirmed that 16HBE14 do not express endogenous SPLUNC1 (Supplementary Figure 4*A*). The presence of WT rSPLUNC1 significantly inhibited Nm adherence to 16HBE14 cells (6.8%; *P* < .0001), compared with BSA (89.67%; Figure 4A; see Supplementary Figure 5). Both the G22E and ΔN mutant rSPLUNC1 proteins elicited a reduced ability to inhibit adherence to 16HBE14 cells by Nm, giving an adherence capacity of 26.2% and 16.3%, respectively, and suggesting an impaired rSPLUNC1 function. Consistent with its effect on adherence, the WT rSPLUNC1 also elicited significant inhibition (0.044%; *P* = .01) on bacterial invasion into 16HEB14 cells (Figure 4B) with respect to BSA (0.097%). Neither mutant rSPLUNC1 protein showed any inhibitory effect on invasion.

### Mutant G22E Recombinant SPLUNC1 Protein Exerts a Dominant-negative Effect

**Figure 5.**
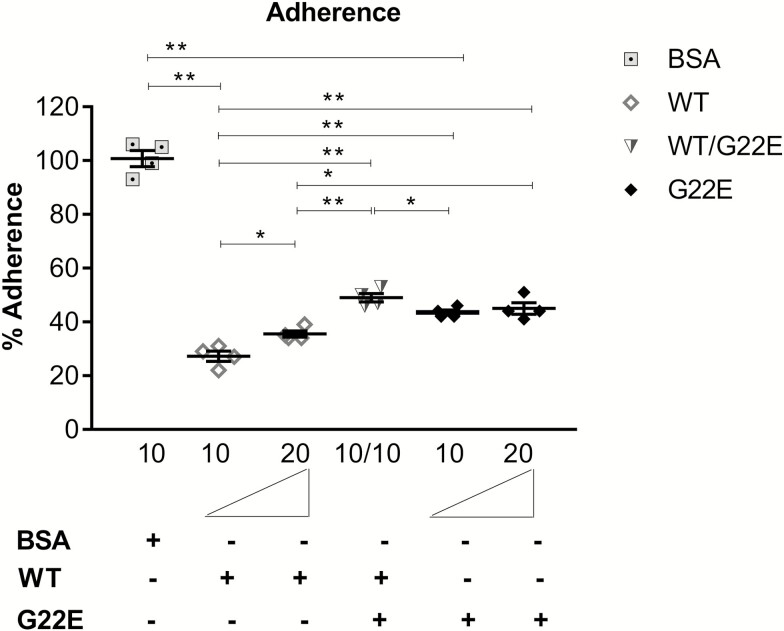
The mutant G22E rSPLUNC1 protein is dominant negative. Bacterial adherence to 16HBE14 was tested in the presence of increasing doses of mutant G22E and/or WT rSPLUNC1. The amounts of recombinant protein used are displayed below the bar. The adherent bacteria were determined by CFU counts. Each condition was carried out in triplicate and the means ± SEMs from 5 independent experiments, following 4 hours adherence, are shown. *P < .05, **P < .001: determined by 1-way ANOVA with Tukey’s test. Abbreviations: 16HBE14, human bronchial epithelial cell line; ANOVA, analysis of variance; rSPLUNC1, recombinant SPLUNC1; SEM, standard error of the mean; WT, wild-type.

We investigated the molecular mechanisms by which the patients’ heterozygous G22E mutation may exert its deleterious effects. We showed that WT rSPLUNC1 is capable of inhibiting adherence and invasion of 16HBE14 cells by Nm at physiologically relevant concentrations [51], whereas the G22E mutant showed attenuation of the rSPLUNC1 function. Thus, we next assessed the effect of the mutant G22E protein on the WT protein function by treating cells with equal amounts of WT and G22E mutant rSPLUNC1 using the bacterial adherence assay. We observed similar levels of inhibition on adherence to 16HBE14 cells by Nm in the presence of 10 and 15 µg/ml of WT rSPLUNC1 (see Supplementary Figure 6). From this, we inferred that the rSPLUNC1 protein operates at a minimal effective dose of 10 µg/ml. We observed less inhibition at 220 vs 10ug/ml of WT rSPLUNC1 (26.8% vs 35.8%), although this was still significantly different from all doses tested using mutant G22E rSPLUNC1 (43.5% and 45.8%; Figure 5; see Supplementary Figure 6). We used the minimal effective dose of WT rSPLUNC1 (10 µg/ml) as the baseline to test any inhibitory effect of the mutant G22E protein. We found that the addition of mutant G22E to WT rSPLUNC1 led to the attenuation of the WT SPLUNC1 function, from 27.3% to 49.0% adherence, resulting in a similar level of bacterial adherence to the G22E mutant alone (45.0%; Figure 5).

### Mutant G22E Recombinant SPLUNC1 is Able to Bind Lipopolysaccharide

**Figure 6.**
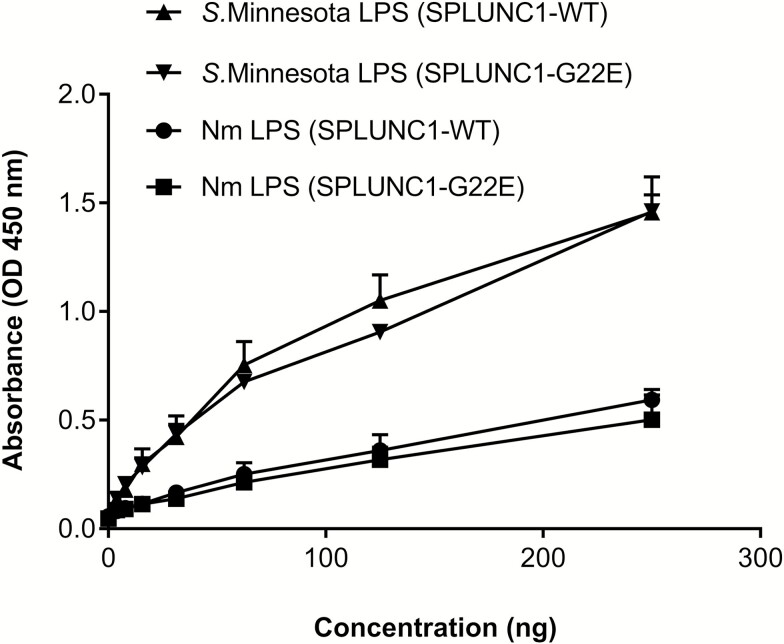
Both WT and mutant G22E rSPLUNC1 proteins bind LPS. Binding of Nm LPS or *Salmonella* Minnesota LPS are shown, with increasing doses of either WT or mutant G22E rSPLUNC1 proteins determined by ELISA. All experiments were performed 3 times and results are shown as means ± SDs. Abbreviations: ELISA, enzyme-linked immunosorbent assay; LPS, lipopolysaccharide; Nm, *Neisseria meningitidis*; OD, optical density; rSPLUNC1, recombinant SPLUNC1; SD, standard deviation; WT, wild-type.

Several studies have demonstrated that SPLUNC1 shares homology with host immune defense neutrophil proteins, such as BPI, and thus retains the ability to bind directly to bacterial LPS [44]. ELISA-based LPS-binding assays were used to evaluate SPLUNC1’s ability to bind LPS produced by Gram-negative bacteria such as Nm. LPS is recognized by human cells expressing TLR4, upon which a cascade of host immune defense mechanisms are initiated [55]. We used Nm LPS from the *IgtB* mutant, which lacks the terminal galactose residue but is not known to impact binding to surface proteins [56]. WT and mutant rSPLUNC1 bound equally well to Nm LPS (Figure 6), as well as to LPS derived from another Gram-negative bacterium: *Salmonella enterica var* Minnesota.

## DISCUSSION

We undertook WES of a cohort of patients with IMD and identified 3 meningococcal patients from 2 families with a novel mutation in *SPLUNC1*. The encoding protein, SPLUNC1, is secreted by airway epithelial cells and its expression has been shown to be highly modulated during bacterial or viral infections [31, 44, 51, 52]. Previous studies have demonstrated that SPLUNC1 is an innate immune defense protein with antimicrobial activity that can impede bacterial growth by modifying biofilm formation and has bacteriostatic activity against Gram-negative bacteria, including *P. aeruginosa* [27, 30]. Given the role of biofilms in meningococcal infections [57, 58], we sought to assess the biofilm-inhibitory property of SPLUNC1 in the context of meningococcal colonization. We have shown that SPLUNC1 inhibits Nm biofilm formation and protects against adherence and subsequent invasion into human 16HBE14 epithelial cells. We further identified a novel missense *SPLUNC1* (p.G22E) mutation by WES of IMD patients, and demonstrated the relevance of SPLUNC1 in human meningococcal infection using in vitro assays.

Our results suggest that SPLUNC1 protects against Nm infection by inhibiting early biofilm formation. To understand how this antibiofilm effect relates to defense against Nm infection of human airway epithelial cells, we assessed Nm adherence and invasion into 16HBE14 epithelial cells using recombinant WT and mutant rSPLUNC1 proteins. Our data show that the presence of rSPLUNC1 results in marked reductions in Nm adherence and subsequent invasion into 16HBE14 cells. Our bacterial growth assays showed that rSPLUNC1 does not elicit a bactericidal effect on meningococcal growth, as we did not detect direct bacterial killing. Thus, the reductions we observed following Nm infection of the human 16HBE14 cells are unlikely to be due to the reduced bacterial burden by direct bacterial killing, but rather resulted through the prevention of Nm biofilm formation or adherence and invasion into epithelial cells. Our findings are consistent with a previous report on SPLUNC1’s surfactant activity on Gram-negative bacteria, including *Klebsiella pneumoniae*, where SPLUNC1 was shown to inhibit bacterial biofilm formation on cultured human cells but had no direct bactericidal effect [27].

To address the question of whether our novel G22E mutation confers susceptibility to IMD, we characterized the mutation in the context of an Nm infection using rSPLUNC1 proteins. We demonstrated that the G22E mutant had an impaired ability to inhibit early Nm biofilm formation, compared with WT rSPLUNC1, when cultured on an abiotic surface. Consistent with this, we observed a significant increase in the number of Nm cells adhering to and invading human 16HBE14 cells in the presence of mutant G22E rSPLUNC1, compared with WT rSPLUNC1. Furthermore, as patient cells expressing the heterozygous mutation were unavailable, the impact of the mutant protein on WT function was tested using the Nm adherence assay. The mutant G22E protein was found to function in a dominant negative manner from this assay [41, 42]. Our results suggest that the G22E mutation located in this functional domain may disrupt the normal function of SPLUNC1 through the modulation of its surfactant properties, including surface tension regulation on epithelial surfaces in a steady state and during infection. Furthermore, the impairment in antibiofilm activity is not caused by a deficiency in the LPS-binding ability of the G22E mutant, as it had normal LPS binding. These findings support the proposal that the LPS-binding and antibiofilm activities of the protein are not functionally linked, and the corresponding domains do not necessarily overlap [42].

In conclusion, we have shown that SPLUNC1 plays an important role in protection against Nm adherence and the subsequent invasion of respiratory epithelial cells. We further demonstrated that the novel G22E mutation identified in 3 IMD patients may confer a predisposition to IMD by impairing the protective role of SPLUNC1 against Nm infection. Taken together, these results build on earlier findings of the antimicrobial role that SPLUNC1 plays in Gram-negative bacterial infections by preventing biofilm formation. We describe here a novel genetic aetiology of IMD in relation to the colonization and invasion of host epithelial barriers by Nm, thereby increasing susceptibility to IMD. Identifying rare mutations that modulate the activity of SPLUNC1 will help unravel the functional domains of this multifunctional, innate immune defense protein and facilitate the identification of individuals who are at risk of developing invasive disease.

## Supplementary Data

Supplementary materials are available at *Clinical Infectious Diseases* online. Consisting of data provided by the authors to benefit the reader, the posted materials are not copyedited and are the sole responsibility of the authors, so questions or comments should be addressed to the corresponding author.

ciz600_Suppl_Supplementary_Material

## Notes

A full list of EULCIDS Consortium in online data supplement.

## Notes

***Acknowledgments.*** The authors thank Peter van der Ley (Institute for Translational Vaccinology, Netherlands) for the generous gift of purified *Neisseria meningitidis* lipopolysaccharide and Beth Holder and Aubrey J. Cunnington for critical reading of the manuscript.

***Financial support.*** This work was supported by the European Seventh Framework Programme for Research and Technological Development (European Union Childhood Life-Threatening Infectious Disease Study [EUCLIDS] Grant Agreement 279185) and in part by European Union’s Horizon 2020 research and innovation programme personalized risk assessment in febrile illness to optimize real-life management across the European Union (Grant Agreement 668303), the National Institutes of Health, National Heart, Lung, and Blood Institute (grant number HL-125128), and the National Institute for Health Research Imperial Biomedical Research Centre at Imperial College Healthcare National Health Service Trust (grant number 70931). V. S.-S. was supported by the Medical Research Foundation.

***Potential conflicts of interest.*** All other authors report no potential conflicts. All authors have submitted the ICMJE Form for Disclosure of Potential Conflicts of Interest. Conflicts that the editors consider relevant to the content of the manuscript have been disclosed.
