# Correction to: A Rare Mutation in SPLUNC1 Affects Bacterial Adherence and Invasion in Meningococcal Disease

**DOI:** 10.1093/cid/ciac263

**Published:** 2022-05-24

An error appeared in the 15 May 2020 issue of the journal [Mashbat F, Bellos E, Hodeib S, et al. A Rare Mutation in SPLUNC1 Affects Bacterial Adherence and Invasion in Meningococcal Disease. Clin Infect Dis 2020; 70(10): 2045-53. https://doi.org/10.1093/cid/ciz600].

Currently the author list on the title page states “Bayarchimeg Mashbat, Evangelos Bellos, Stephanie Hodeib, Fadil Bidmos, Ryan S. Thwaites, Yaxuan Lu, Victoria Wright, Jethro Herberg, Daniela S. Klobassa, Werner Zenz, Trevor T. Hansel, Simon Nadel, Paul R. Langford, Luregn J. Schlapbach, Ming-Shi Li, Matthew Redinbo, Y. Peter Di, Michael Levin and Vanessa Sancho-Shimizu”

The correct author list, adding William G. Walton, should be as follows: “Bayarchimeg Mashbat, Evangelos Bellos, Stephanie Hodeib, Fadil Bidmos, Ryan S. Thwaites, Yaxuan Lu, Victoria Wright, Jethro Herberg, Daniela S. Klobassa, William G. Walton, Werner Zenz, Trevor T. Hansel, Simon Nadel, Paul R. Langford, Luregn J. Schlapbach, Ming-Shi Li, Matthew Redinbo, Y. Peter Di, Michael Levin and Vanessa Sancho-Shimizu”

The author regrets this error.

