# Supplementary material for: A Rare Mutation in SPLUNC1 Affects Bacterial Adherence and Invasion in Meningococcal Disease
Source: Clin Infect Dis. 2019 Jul 1;70(10):2045–53. doi: 10.1093/cid/ciz600 (PMC7201419; doi:10.1093/cid/ciz600)
Supplement: ciz600_Suppl_Supplementary_Material [file ciz600_suppl_supplementary_material.pdf]

Online Data Supplement

A novel mutation in *SPLUNC1* underlies meningococcal disease affecting bacterial adherence and invasion

Bayarchimeg Mashbat[1], Evangelos Bellos[1], Stephanie Hodeib[1], Fadil Bidmos[1], Ryan S. Thwaites[2], Yaxuan Lu[1], Victoria J. Wright[1], Jethro A. Herberg[1], Daniela S. Klobassa[3], Werner Zenz[3], Trevor T. Hansel[2], Peter Openshaw[2], Simon Nadel[4], Paul R. Langford[1], Luregn J. Schlapbach[5,6,7,8], Ming-Shi Li[1], Matthew R. Redinbo[9,10,11], Y. Peter Di[12], Michael Levin[1] and Vanessa Sancho-Shimizu[1,13,] on behalf of EUCLIDS Consortium*.

[1]Department of Paediatric infectious diseases, Division of Medicine, Imperial College London, Norfolk Place, UK.
[2] National Heart and Lung Institute, Imperial College London, UK.[3] Department of Pediatric and Adolescence Surgery, Division of General Pediatric Surgery, Medical University Graz, Austria
4 Paediatric Intensive Care Unit, St. Mary's Hospital, Imperial College Healthcare Trust, London, UK.[5]Faculty of Medicine, The University of Queensland, Brisbane, Australia
[6]Paediatric Critical Care Research Group, The University of Queensland, Brisbane, Australia
[7]Paediatric Intensive Care Unit, Lady Cilento Children's Hospital, Children's Health Queensland, Brisbane, Australia
[8]Department of Pediatrics, Bern University Hospital, Inselspital, University of Bern, Bern, Switzerland
[9]Department of Chemistry, University of North Carolina, Chapel Hill, NC 27599, USA
[10]Department of Biochemistry and Biophysics, University of North Carolina, Chapel Hill, NC 27599, USA.
[11]Lineberger Comprehensive Cancer Center, University of North Carolina, Chapel Hill, NC 27599, USA
[12]Department of Environmental and Occupational Health, University of Pittsburgh, Pennsylvania, USA.
[13]Departments of Virology, Division of Medicine, Imperial College London, Norfolk Place, UK.

Corresponding author details:

Vanessa Sancho-Shimizu Ph.D.
Department of Paediatric infectious diseases, St Mary's Medical School Building
Imperial College London, Norfolk Place, London W2 1PG
Tel: ++44 (0)20 7594 3914
Fax: ++44 (0)20 7594 3984
E-mail: v.sancho-shimizu@imperial.ac.uk

***EUCLIDS consortium (www.euclids-project.eu) is composed by:**

Imperial College partner (UK)

Members of the EUCLIDS Consortium at Imperial College London (UK)

Principal and co-investigators

<u>Michael Levin (grant application, EUCLIDS Coordinator)</u>
Dr. Lachlan Coin (bioinformatics)
Stuart Gormley (clinical coordination)
Shea Hamilton (proteomics)
Jethro Herberg (grant application, PI)
Bernardo Hourmat (project management)
Clive Hoggart (statistical genomics)
Myrsini Kaforou (bioinformatics)
Vanessa Sancho-Shimizu (genetics)
Victoria Wright (grant application, scientific coordination)

<u>Consortium members at Imperial College</u>
Amina Abdulla
Paul Agapow
Maeve Bartlett
Evangelos Bellos
Hariklia Eleftherohorinou
Rachel Galassini
David Inwald
Bayarchimeg Mashbat
Stefanie Menikou
Sobia Mustafa
Simon Nadel
Rahmeen Rahman
Clare Thakker

EUCLIDS UK Clinical Network
Poole Hospital NHS Foundation Trust, Poole: Dr S Bokhandi (PI), Sue Power, Heather Barham
Cambridge University Hospitals NHS Trust, Cambridge: Dr N Pathan (PI), Jenna Ridout, Deborah White, Sarah Thurston
University Hospital Southampton, Southampton: Prof S Faust (PI), Dr S Patel (co-investigator), Jenni McCorkell.
Nottingham University Hospital NHS Trust: Dr P Davies (PI), Lindsey Crate, Helen Navarra, Stephanie Carter
University Hospitals of Leicester NHS Trust, Leicester: Dr R Ramaiah (PI), Rekha Patel
Portsmouth Hospitals NHS Trust, London: Dr Catherine Tuffrey (PI), Andrew Gribbin, Sharon McCready
Great Ormond Street Hospital, London: Dr Mark Peters (PI), Katie Hardy, Fran Standing, Lauren O'Neill, Eugenia Abelake
King's College Hospital NHS Foundation Trust, London; Dr Akash Deep (PI), Eniola Nsirim
Oxford University Hospitals NHS Foundation Trust, Oxford Prof A Pollard (PI), Louise Willis, Zoe Young

Kettering General Hospital NHS Foundation Trust, Kettering: Dr C Royad (PI), Sonia White
Central Manchester NHS Trust, Manchester: Dr PM Fortune (PI), Phil Hudnott

SERGAS Partner (Spain)

Principal Investigators
Federico Martinón-Torres[1]
Antonio Salas[1,2]

GENVIP RESEARCH GROUP (in alphabetical order):
Fernando Álvez González[1], Ruth Barral-Arca[1,2], Miriam Cebey-López[1], María José Curras-Tuala[1,2], Natalia García[1], Luisa García Vicente[1], Alberto Gómez-Carballa[1,2], Jose Gómez Rial[1], Andrea Grela Beiroa[1], Antonio Justicia Grande[1], Pilar Leboráns Iglesias[1], Alba Elena Martínez Santos[1], Federico Martinón-Torres[1], Nazareth Martinón-Torres[1], José María Martinón Sánchez[1], Beatriz Morillo Gutiérrez[1], Belén Mosquera Pérez[1], Pablo Obando Pacheco[1], Jacobo Pardo-Seco[1,2], Sara Pischedda[1,2], Irene Rivero Calle[1], Carmen Rodríguez-Tenreiro[1], Lorenzo Redondo-Collazo[1], Antonio Salas Ellacuriaga[1,2], Sonia Serén Fernández[1], María del Sol Porto Silva[1], Ana Vega[1,3,] Lucía Vilanova Trillo[1].

[1] Translational Pediatrics and Infectious Diseases, Pediatrics Department, Hospital Clínico Universitario de Santiago, Santiago de Compostela, Spain, and GENVIP Research Group (www.genvip.org), Instituto de Investigación Sanitaria de Santiago, Galicia, Spain.
[2] Unidade de Xenética, Departamento de Anatomía Patolóxica e Ciencias Forenses, Instituto de Ciencias Forenses, Facultade de Medicina, Universidade de Santiago de Compostela, and GenPop Research Group, Instituto de Investigaciones Sanitarias (IDIS), Hospital Clínico Universitario de Santiago, Galicia, Spain
[3] Fundación Pública Galega de Medicina Xenómica, Servizo Galego de Saúde (SERGAS), Instituto de Investigaciones Sanitarias (IDIS), and Grupo de Medicina Xenómica, Centro de Investigación Biomédica en Red de Enfermedades Raras (CIBERER), Universidade de Santiago de Compostela (USC), Santiago de Compostela, Spain

***EUCLIDS SPANISH CLINICAL NETWORK**:*
Susana Beatriz Reyes[1], María Cruz León León[1], Álvaro Navarro Mingorance[1], Xavier Gabaldó Barrios[1], Eider Oñate Vergara[2], Andrés Concha Torre[3], Ana Vivanco[3], Reyes Fernández[3], Francisco Giménez Sánchez[4], Miguel Sánchez Forte[4], Pablo Rojo[5], J.Ruiz Contreras[5], Alba Palacios[5], Cristina Epalza Ibarrondo[5], Elizabeth Fernández Cooke[5], Marisa Navarro[6], Cristina Álvarez Álvarez[6], María José Lozano[6], Eduardo Carreras[7], Sonia Brió Sanagustín[7], Olaf Neth[8], Mª del Carmen Martínez Padilla[9], Luis Manuel Prieto Tato[10], Sara Guillén[10], Laura Fernández Silveira[11], David Moreno[12].

[1] Hospital Clínico Universitario Virgen de la Arrixaca; Murcia, Spain.
[2] Hospital de Donostia; San Sebastián, Spain.
[3] Hospital Universitario Central de Asturias; Asturias, Spain.
[4] Complejo Hospitalario Torrecárdenas; Almería, Spain.
[5] Hospital Universitario 12 de Octubre; Madrid, Spain.

[6] Hospital General Universitario Gregorio Marañón; Madrid, Spain.
[7] Hospital de la Santa Creu i Sant Pau; Barcelona, Spain.
[8] Hospital Universitario Virgen del Rocío; Sevilla, Spain.
[9] Complejo Hospitalario de Jaén; Jaén, Spain.
[10] Hospital Universitario de Getafe; Madrid, Spain.
[11] Hospital Universitario y Politécnico de La Fe; Valencia, Spain.
[12] Hospital Regional Universitario Carlos Haya; Málaga, Spain.

Members of the Pediatric Dutch Bacterial Infection Genetics (PeD-BIG) network (the Netherlands)

Steering committee:
Coordination: R. de Groot [1], A.M. Tutu van Furth [2], M. van der Flier [1]
Coordination Intensive Care: N.P. Boeddha [3], G.J.A. Driessen [3], M. Emonts [3, 4, 5], J.A. Hazelzet [3]
Other members: T.W. Kuijpers [7], D. Pajkrt [7], E.A.M. Sanders [6] , D. van de Beek [8], A. van der Ende [8]
Trial coordinator: H.L.A. Philipsen [1]

Local investigators (in alphabetical order)
A.O.A. Adeel [9], M.A. Breukels [10], D.M.C. Brinkman [11], C.C.M.M. de Korte [12], E. de Vries [13], W.J. de Waal [15], R. Dekkers [15], A. Dings-Lammertink [16], R.A. Doedens [17], A.E. Donker [18], M. Dousma[19], T.E. Faber [20], G.P.J.M. Gerrits[21], J.A.M. Gerver [22], J. Heidema [23], J. Homan-van der Veen [24], M.A.M. Jacobs [25], N.J.G. Jansen [6], P. Kawczynski [26], K. Klucovska [27], M.C.J. Kneyber [28], Y. Koopman-Keemink [29], V.J. Langenhorst [30], J. Leusink [31], B.F. Loza [32], I.T. Merth [33], C.J. Miedema [34], C. Neeleman [1], J.G. Noordzij [35], C.C. Obihara [36], A.L.T. van Overbeek – van Gils [37], G.H. Poortman [38],S.T. Potgieter [39], J. Potjewijd [40], P.P.R. Rosias [41], T. Sprong [21], G.W. ten Tussher [42], B.J. Thio [43], G.A. Tramper-Stranders [44], M. van Deuren [1], H. van der Meer [2], A.J.M. van Kuppevelt [45], A.M. van Wermeskerken [46], W.A. Verwijs [47], T.F.W. Wolfs [4].

1. Radboud University Medical Center – Amalia Children’s Hospital, Nijmegen, The Netherlands
2. Vrije Universiteit University Medical Center, Amsterdam, The Netherlands
3. Erasmus Medical Center – Sophia Children’s Hospital, Rotterdam, The Netherlands
4. Institute of Cellular Medicine, Newcastle University, Newcastle upon Tyne, United Kingdom
5. Paediatric Infectious Diseases and Immunology Department, Newcastle upon Tyne Hospitals Foundation Trust, Great North Children's Hospital, Newcastle upon Tyne, United Kingdom
6. University Medical Center Utrecht – Wilhelmina Children’s Hospital, Utrecht, The Netherlands
7. Academic Medical Center – Emma Children’s Hospital, University of Amsterdam, Amsterdam, The Netherlands
8. Academic Medical Center, University of Amsterdam, Amsterdam, The Netherlands
9. Kennemer Gasthuis, Haarlem, The Netherlands
10. Elkerliek Hospital, Helmond, The Netherlands
11. Alrijne Hospital, Leiderdorp, The Netherlands
12. Beatrix Hospital, Gorinchem, The Netherlands

13. Jeroen Bosch Hospital, ‘s-Hertogenbosch, The Netherlands
14. Diakonessenhuis, Utrecht, The Netherlands
15. Maasziekenhuis Pantein, Boxmeer, The Netherlands
16. Gelre Hospitals, Zutphen, The Netherlands
17. Martini Hospital, Groningen, The Netherlands
18. Maxima Medical Center, Veldhoven, The Netherlands
19. Gemini Hospital, Den Helder, The Netherlands
20. Medical Center Leeuwarden, Leeuwarden, The Netherlands
21. Canisius-Wilhelmina Hospital, Nijmegen, The Netherlands
22. Rode Kruis Hospital, Beverwijk, The Netherlands
23. St. Antonius Hospital, Nieuwegein, The Netherlands
24. Deventer Hospital, Deventer, The Netherlands
25. Slingeland Hospital, Doetinchem, The Netherlands
26. Refaja Hospital, Stadskanaal, The Netherlands
27. Bethesda Hospital, Hoogeveen, The Netherlands
28. University Medical Center Groningen, Beatrix Children’s hospital, Groningen, The Netherlands
29. Haga Hospital – Juliana Children’s Hospital, Den Haag, The Netherlands
30. Isala Hospital, Zwolle, The Netherlands
31. Bernhoven Hospital, Uden, The Netherlands
32. VieCuri Medical Center, Venlo, The Netherlands
33. Ziekenhuisgroep Twente, Almelo-Hengelo, The Netherlands
34. Catharina Hospital, Eindhoven, The Netherlands
35. Reinier de Graaf Gasthuis, Delft, The Netherlands
36. ETZ Elisabeth, Tilburg, The Netherlands
37. Scheper Hospital, Emmen, The Netherlands
38. St. Jansdal Hospital, Hardewijk, The Netherlands
39. Laurentius Hospital, Roermond, The Netherlands
40. Isala Diaconessenhuis, Meppel, The Netherlands
41. Zuyderland Medical Center, Sittard-Geleen, The Netherlands
42. Westfriesgasthuis, Hoorn, The Netherlands
43. Medisch Spectrum Twente, Enschede, The Netherlands
44. St. Franciscus Gasthuis, Rotterdam, The Netherlands
45. Streekziekenhuis Koningin Beatrix, Winterswijk, The Netherlands
46. Flevo Hospital, Almere, The Netherlands
47. Zuwe Hofpoort Hospital, Woerden, The Netherlands

Swiss Pediatric Sepsis Study

Steering Committee*: Luregn J Schlapbach, MD, FCICM [1,2,3], Philipp Agyeman, MD [1], Christoph Aebi, MD [1], Christoph Berger, MD [1]*

Investigators: Luregn J Schlapbach, MD, FCICM [1,2,3], Philipp Agyeman, MD [1], Christoph Aebi, MD [1], Eric Giannoni, MD [4,5], Martin Stocker, MD [6], Klara M Posfay-Barbe, MD [7], Ulrich Heininger, MD [8], Sara Bernhard-Stirnemann, MD [9], Anita Niederer-Loher, MD [10], Christian Kahlert, MD [10], Paul Hasters, MD [11], Christa Relly, MD [12], Walter Baer, MD [13], Alessandro Borghesi PhD[14,15,16], MD PhD, Samira Asgari PhD PhD[14,15,17,18], Christoph Berger, MD [12] **for the Swiss Pediatric Sepsis Study**

[1.] Department of Pediatrics, Inselspital, Bern University Hospital, University of Bern, Switzerland
[2.] Paediatric Critical Care Research Group, Mater Research Institute, University of Queensland, Brisbane, Australia
[3.] Paediatric Intensive Care Unit, Lady Cilento Children's Hospital, Children's Health Queensland, Brisbane, Australia
[4.] Service of Neonatology, Lausanne University Hospital, Lausanne, Switzerland
[5.] Infectious Diseases Service, Lausanne University Hospital, Lausanne, Switzerland
[6.] Department of Pediatrics, Children's Hospital Lucerne, Lucerne, Switzerland
[7.] Pediatric Infectious Diseases Unit, Children's Hospital of Geneva, University Hospitals of Geneva, Geneva, Switzerland
[8.] Infectious Diseases and Vaccinology, University of Basel Children's Hospital, Basel, Switzerland
[9.] Children's Hospital Aarau, Aarau, Switzerland
[10.] Division of Infectious Diseases and Hospital Epidemiology, Children's Hospital of Eastern Switzerland St. Gallen, St. Gallen, Switzerland
[11.] Department of Neonatology, University Hospital Zurich, Zurich, Switzerland
[12.] Division of Infectious Diseases and Hospital Epidemiology, and Children's Research Center, University Children's Hospital Zurich, Switzerland
[13.] Children's Hospital Chur, Chur, Switzerland
[14.] Swiss Institute of Bioinformatics, Lausanne, Switzerland
[15.] Global Health Institute, School of Life Sciences, École Polytechnique Fédérale de Lausanne (EPFL), Lausanne, Switzerland.
[16.] Neonatal Intensive Care Unit, Fondazione IRCCS Policlinico San Matteo, Pavia, Italy.
[17.] Brigham and Women's Hospital, Harvard Medical School, Boston, Massachusetts, USA.
[18.] Broad Institute of Harvard and MIT, Cambridge, Massachusetts, USA.

Liverpool Partner

Principal Investigators
Enitan D Carrol[1]
Stéphane Paulus [1,2]

ALDER HEY SERIOUS PAEDIATRIC INFECTION RESEARCH GROUP (ASPIRE) (in alphabetical order):
Hannah Frederick[3], Rebecca Jennings[3], Joanne Johnston[3], Rhian Kenwright[3]

[1] Department of Clinical Infection, Microbiology and Immunology, University of Liverpool Institute of Infection and Global Health , Liverpool, England
[2] Alder Hey Children's Hospital, Department of Infectious Diseases, Eaton Road, Liverpool, L12 2AP
[3] Alder Hey Children's Hospital, Clinical Research Business Unit, Eaton Road, Liverpool, L12 2AP

Micropathology Ltd

Colin G Fink[1,2], Elli Pinnock[1]

[1]Micropathology Ltd Research and Diagnosis
[2]University of Warwick

Newcastle partner

Principle Investigator
Marieke Emonts[1,2]
Co-Investigator
Rachel Agbeko[1,3]
[1] Institute of Cellular Medicine, Newcastle University, Newcastle upon Tyne, United Kingdom
[2] Paediatric Infectious Diseases and Immunology Department, Newcastle upon Tyne Hospitals Foundation Trust, Great North Children's Hospital, Newcastle upon Tyne, United Kingdom
[3] Paediatric Intensive Care Unit, Newcastle upon Tyne Hospitals Foundation Trust, Great North Children's Hospital, Newcastle upon Tyne, United Kingdom

Gambia partner

Suzanne Anderson: Principal Investigator and West African study oversight:
Fatou Secka: Clinical research fellow and study co-ordinator
Additional Gambia site team (consortium members):
Kalifa Bojang: co-PI
Isatou Sarr: Senior laboratory technician
Ngane Kebbeh: Junior laboratory technician
Gibbi Sey: lead research nurse Medical Research Council Clinic
Momodou Saidykhan: lead research nurse Edward Francis Small Teaching Hospital
Fatoumatta Cole: Data manager
Gilleh Thomas: Data manager
Martin Antonio: Local collaborator

Medical Research Council Unit Gambia
PO Box 273
Banjul
The Gambia

Austrian partner

PI: Werner Zenz[1]
Co-Investigators/Steering committee:
Daniela S. Klobassa[1], Alexander Binder[1], Nina A. Schweintzger[1], Manfred Sagmeister[1]
[1]University Clinic of Paediatrics and Adolescent Medicine, Department of General Paediatrics, Medical University Graz, Austria

Austrian network, participating centres in Austria, Germany, Italy, Serbia, Lithuania, patient recruitment (in alphabetical order):

Hinrich Baumgart[1], Markus Baumgartner[2], Uta Behrends[3], Ariane Biebl[4], Robert Birnbacher[5], Jan-Gerd Blanke[6], Carsten Boelke[7], Kai Breuling[3], Jürgen Brunner[8], Maria Buller[9], Peter Dahlem[10], Beate Dietrich[11], Ernst Eber[12], Johannes Elias[13], Josef Emhofer[2], Rosa Etschmaier[14], Sebastian Farr[15], Ylenia Girtler[16], Irina Grigorow[17], Konrad Heimann[18], Ulrike Ihm[19], Zdenek Jaros[20], Hermann Kalhoff[21], Wilhelm Kaulfersch[22], Christoph Kemen[23], Nina Klocker[24], Bernhard Köster[25], Benno Kohlmaier[26], Eleni Komini[27], Lydia Kramer[3], Antje Neubert[28], Daniel Ortner[29], Lydia Pescollderungg[16], Klaus Pfurtscheller[30], Karl Reiter[31], Goran Ristic[32], Siegfried Rödl[30], Andrea Sellner[26], Astrid Sonnleitner[26], Matthias Sperl[33], Wolfgang Stelzl[34], Holger Till[1], Andreas Trobisch[26], Anne Vierzig[35], Ulrich Vogel[12], Christina Weingarten[36], Stefanie Welke[37], Andreas Wimmer[38], Uwe Wintergerst[39], Daniel Wüller[40], Andrew Zaunschirm[41], Ieva Ziuraite[42], Veslava Žukovskaja[42]

[1]Department of Pediatric and Adolescence Surgery, Division of General Pediatric Surgery, Medical University Graz, Austria
[2]Department of Pediatrics, General Hospital of Steyr, Austria
[3]Department of Pediatrics/Department of Pediatric Surgery, Technische Universität München (TUM), Munich, Germany
[4]Department of Pediatrics, Kepler University Clinic, Medical Faculty of the Johannes Kepler University, Linz, Austria
[5]Department of Pediatrics and Adolesecent Medicine LKH Villach, Austria
[6]Department of Pediatrics and Adolescent Medicine and Neonatology, Hospital Ludmillenstift, Meppen, Germany
[7]Hospital for Children's and Youth Medicine, Oberschwabenklinik, Ravensburg, Germany
[8]Department of Pediatrics, Medical University Innsbruck, Austria
[9]Clinic for Paediatrics and Adolescents Medicine, Sana Hanse-Klinikum Wismar, Germany
[10]Departement of Pediatrics, Medical Center Coburg, Germany
[11]University Medicine Rostock, Department of Pediatrics (UKJ), Rostock, Germany
[12]Department of Pulmonology, Medical University Graz, Austria
[13]Institute for Hygiene and Microbiology, University of Würzburg, Germany
[14]Clinical Institute of Medical and Chemical Laboratory Diagnostics, Medical University Graz, Austria
[15]Department of Pediatric Orthopedics and Adult Foot and Ankle Surgery, Orthopedic Hospital Speising, Vienna, Austria
[16]Department of Paediatrics, Regional Hospital Bolzano, Italy
[17]Department of Pediatrics and Adolescent Medicine, General Hospital Hochsteiermark/Leoben, Austria
[18]Department of Neonatology and Paediatric Intensive Care, Children's University Hospital, RWTH Aachen, Germany
[19]Paediatric Intensive Care Unit, Department of Paediatric Surgery, Donauspital Vienna, Austria
[20]Department of Pediatrics, General Public Hospital, Zwettl, Austria
[21]Pediatric Clinic Dortmund, Germany
[22]Department of Pediatrics and Adolescent Medicine, Klinikum Klagenfurt am Wörthersee, Klagenfurt, Austria
[23]Catholic Children's Hospital Wilhelmstift, Department of Pediatrics, Hamburg, Germany
[24]Department of Pediatrics, Krankenhaus Dornbirn, Austria

[25]Children’s Hospital Luedenscheid, Maerkische Kliniken, Luedenscheid, Germany
[26]Department of General Paediatrics, Medical University Graz, Austria
[27]Department of Paediatrics, Schwarzwald-Baar-Hospital, Villingen-Schwenningen, Germany
[28]Department of Paediatrics and Adolescents Medicine, University Hospital Erlangen, Germany
[29]Department of Pediatrics and Adolescent Medicine, Medical University of Salzburg, Austria
[30]Paediatric Intensive Care Unit, Medical University Graz, Austria
[31]Dr. von Hauner Children's Hospital, Ludwig-Maximilians- Universitaet, Munich, Germany
[32]Mother and Child Health Care Institute of Serbia, Belgrade, Serbia
[33]Department of Pediatric and Adolescence Surgery, Division of Pediatric Orthopedics, Medical University Graz, Austria
[34]Department of Pediatrics, Academic Teaching Hospital, Landeskrankenhaus Feldkirch, Austria
[35]University Children’s Hospital, University of Cologne, Germany
[36]Department of Pediatrics and Adolescent Medicine Wilheminenspital, Vienna, Austria
[37]Department of Pediatric Surgery, Municipal Hospital Karlsruhe, Germany
[38]Hospital of the Sisters of Mercy Ried, Department of Pediatrics and Adolescent Medicine, Ried, Austria
[39]Hospital St. Josef, Braunau, Austria
[40]Christophorus Kliniken Coesfeld Clinic for Pediatrics, Coesfeld, Germany
[41]Department of Paediatrics, University Hospital Krems, Karl Landsteiner University of Health Sciences, Krems, Austria
[42]Children‘s Hospital, Affiliate of Vilnius University Hospital Santariskiu Klinikos, Lithuania

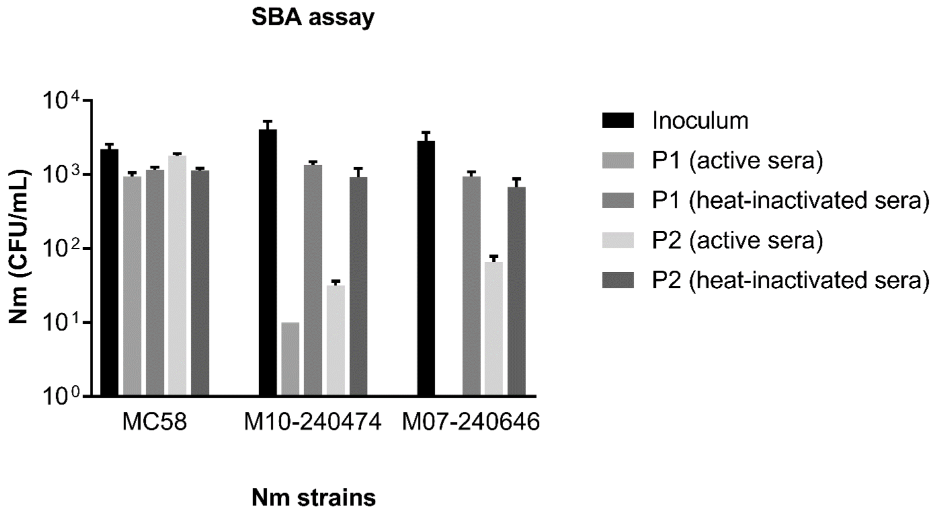

Supplementary Figure 1. Serum bactericidal assay (SBA) of P1 and P2. Serum from P1 and P2 have functional complement and serogroup specific neutralising antibody against Nm serogroup B (MC58, M10-240474 and M07-240646) strains.

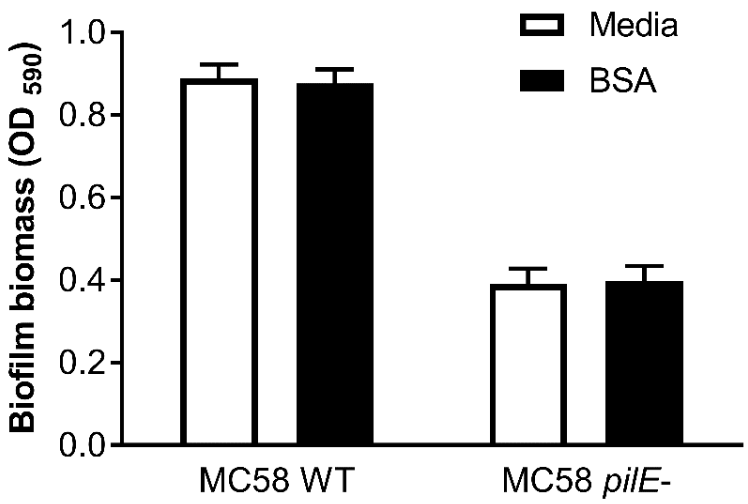

Supplementary Figure 2. BSA does not affect Nm biofilm formation. Biofilm biomass formed by wild-type (WT) or pili deficient (*pilE*-) mutant Nm in the presence of BSA or culture media on a microtitre plate visualised with crystal violet staining. The results are means ± SEM from three independent experiments.

A.

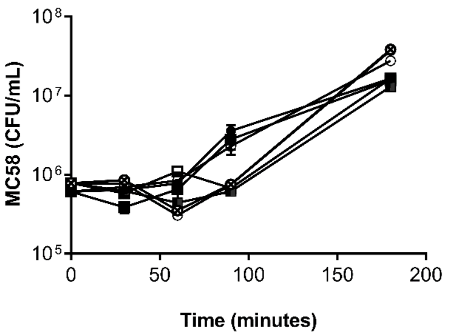

B.

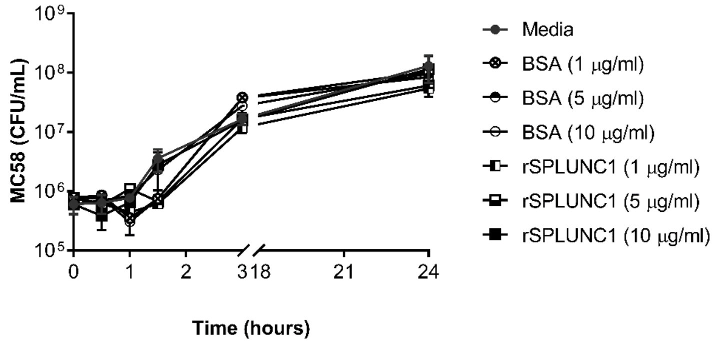

Supplementary Figure 3. Dose response analysis of rSPLUNC1 on Nm growth. Nm was grown in the presence of increasing doses of wild-type (WT) rSPLUNC1 (1, 5, 10 µg/mL) protein or BSA (1, 5, 10 µg/ml) for up to 3 hours (A) or 24 hours (B). Bacterial viability was assessed by counting CFUs. Results are means ± SD from two independent experiments carried out in triplicates. Statistical significance assessed using student's t-test.

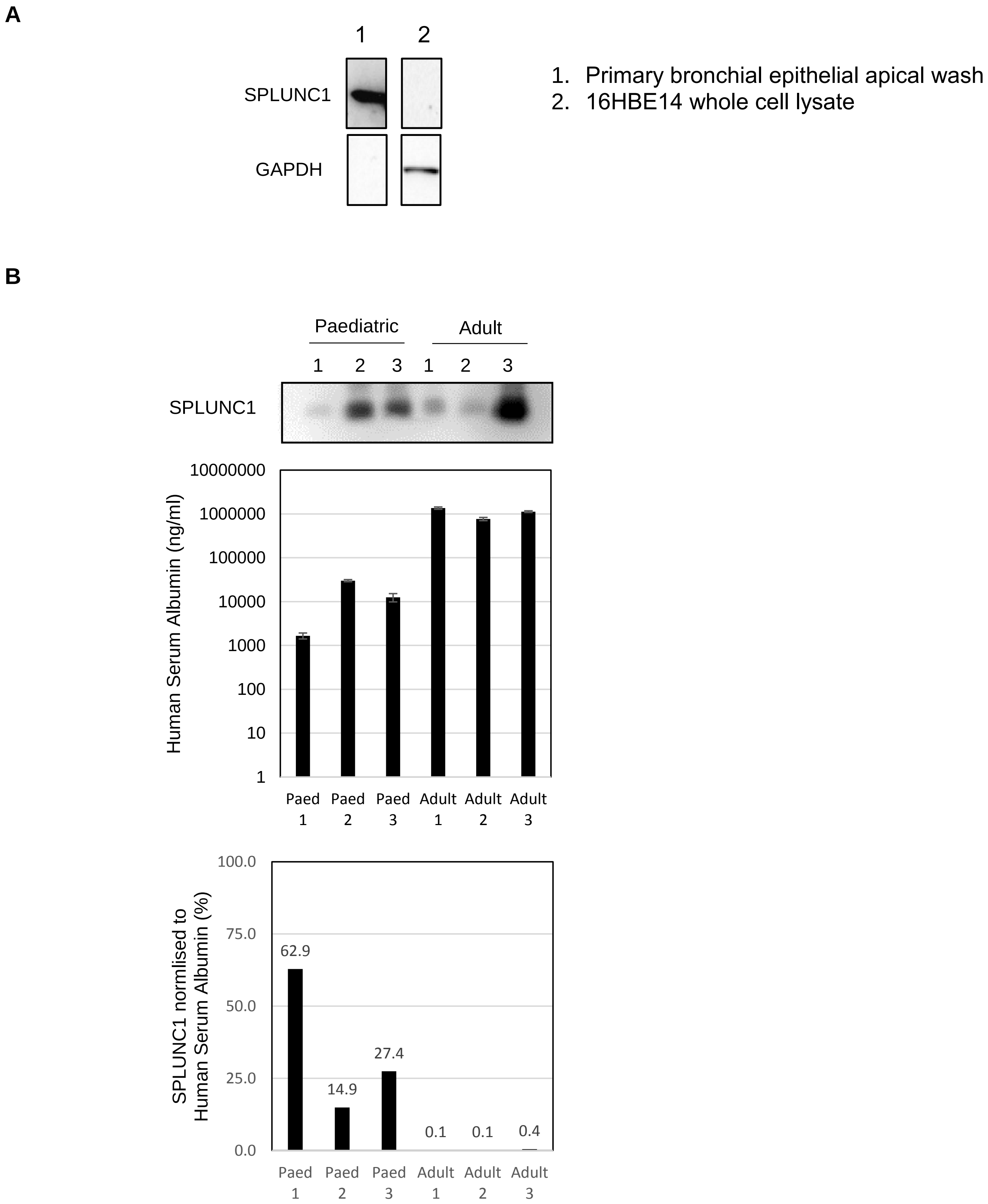
A
1
2
SPLUNC1
GAPDH
1. Primary bronchial epithelial apical wash
2. 16HBE14 whole cell lysate
B
Paediatric
Adult
1 2 3 1 2 3
SPLUNC1
Human Serum Albumin (ng/ml)
10000000
1000000
100000
10000
1000
100
10
1
Paed 1
Paed 2
Paed 3
Adult 1
Adult 2
Adult 3
SPLUNC1 normlised to Human Serum Albumin (%)
100.0
75.0
50.0
25.0
0.0
62.9
14.9
27.4
0.1
0.1
0.4
Paed 1
Paed 2
Paed 3
Adult 1
Adult 2
Adult 3

Supplementary Figure 4: SPLUNC1 protein expression in 16HBE14 cells and in human nasal lining fluid. (A) Whole cell lysate from human bronchial epithelial cell line, 16HBE14 (lane 2) was resolved on a SDS-PAGE gel and immunoblotted for human SPLUNC1. Apical PBS wash from primary human epithelial cells (lane 1) cultured in air liquid interface, known to secrete SPLUNC1, was included as a positive control. GAPDH was used as a loading control. **(B)** SPLUNC1 immunoblot of nasal lining fluid collected from healthy children and adults. Human serum albumin levels were assessed by ELISA to control for protein load. SPLUNC1 levels were normalised to Human Serum Albumin levels and expressed as a relative percentage expression.

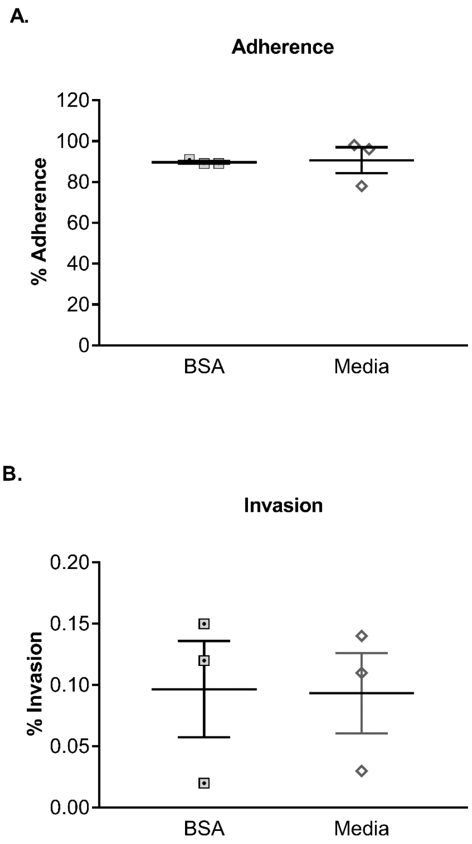

Supplementary Figure 5. BSA does not affect adherence to and invasion of human 16HBE14 cells by Nm. The effect of BSA on NM adherence (**A**) or invasion (**B**) into human 16HBE14 cells was assessed by CFU counts following 4 hours incubation is shown. Each condition was carried out in triplicate and the means (± SEM) from at least three independent experiments are shown.

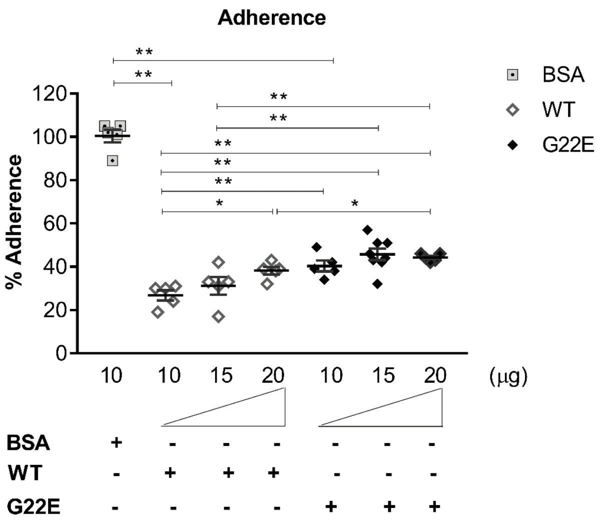

Supplementary Figure 6. Dosage dependent effect of rSPLUNC1 on Nm adherence. Bacterial adherence to human 16HBE14 cells was tested in the presence of increasing doses of WT or G22E mutant rSPLUNC1. The amounts of recombinant protein used are displayed below the bar. The adherent bacteria were determined by CFU counts. Each condition was carried out in triplicate and the means (± SEM) from at least three independent experiments are shown following 4 hours adherence is shown. *$P <$ 0.05, **$P < 0.001$ determined by one-way ANOVA with Tukey's test.

Supplementary Table 1. Summary of patient clinical symptoms and genotype

Patient	Relationship	Age at presentation	Sex	Ethnic Origin	Clinical disease type	Nm serogroup	Mechanical ventilation	Days on ventilation	Days on PICU	Survived	Genotype
P1	Index case	10	F	Caucasian	Meningococcal meningitis and septicaemia	C	Yes	unknown	unknown	Yes	WT/G22E
P2	Affected sibling of P1	2	M	Caucasian	Meningococcal meningitis and septicaemia	unknown	No	unknown	unknown	Yes	WT/G22E
P3	Index case	2	F	Caucasian	Meningococcal septicaemia	B	Yes	8	9	Yes	WT/G22E

Supplementary Table 2. Annotation of rare and shared IBD variants between P1 and P2.

Chromosome	Position (hg17)	Ref	Alt	Gene	Genotype	MAF (EXaC)	Functional impact	CADD Raw Score	CADD Phred Score	BSIFT Score	GDI score	GDI Phred score	Gene damage prediction
							Shared homozygous IBD variants						
1	33430102	T	G	*RNF19B*	1/1	.	missense	0.369	6.343	-0.32	65.38	1.66	Medium
3	14105882	C	A	*TPRXL*	1/1	.	missense	1.146	11.460	-0.12	3182.27	10.74	Medium
10	118231360	G	A	*PNLIPRP3*	1/1	.	missense	-0.820	0.039	-0.02	2278.94	8.83	Medium
14	106329435	T	G	*IGHJ6*	1/1	.	missense	-0.717	0.066	-	N/A	N/A	N/A
16	84224967	G	A	*ADAD2*	1/1	.	missense	-1.895	0.002	0.64	3256.58	10.86	Medium
							Shared novel IBD variants						
1	111957583	A	G	*OVGP1*	0/1	.	missense	-1.910	0.002	-0.43	2799.27	9.99	Medium
1	26608843	C	A	*UBXN11*	0/1	.	missense	3.077	22.500	-0.91	1513.40	7.23	Medium
3	13612457	A	G	*FBLN2*	0/1	.	missense	3.707	23.300	-0.97	754.77	5.45	Medium
4	144619434	C	A	*FREM3*	0/1	.	missense	3.975	23.600	-1.00	2763.74	9.92	Medium
7	142498897	A	G	*TRBC2*	0/1	.	missense	-1.262	0.005	-	N/A	N/A	N/A
8	12612694	G	A	*LONRF1*	0/1	.	missense	4.622	24.500	-0.65	173.36	2.87	Medium
8	144644156	C	T	*GSDMD*	0/1	.	missense	3.368	22.900	-0.99	230.01	3.28	Medium
9	135205330	T	G	*SETX*	0/1	.	missense	4.063	23.700	-0.63	1619.58	7.44	Medium
9	113547938	A	G	*MUSK*	0/1	.	missense	5.580	26.500	-0.96	1294.32	6.77	Medium
12	112669449	G	A	*HECTD4*	0/1	.	missense	6.705	32.000	-0.99	278.36	3.58	Medium
14	75323627	C	A	*PROX2*	0/1	.	missense	4.033	23.700	-0.98	256.61	3.45	Medium
15	60747553	G	A	*-RG2*	0/1	.	missense	3.670	23.300	-0.08	121.32	2.40	Medium
16	81094954	T	C	*C16orf46*	0/1	.	missense	-1.135	0.009	0.02	N/A	N/A	N/A
16	28993322	G	A	*SPNS1*	0/1	.	missense	3.176	22.700	-0.77	238.00	3.33	Medium
16	28883214	C	G	*SH2B1*	0/1	.	missense	5.347	25.900	-1.00	1577.15	7.35	Medium
17	36352489	A	C	*RP11-1407O15.2*	0/1	.	missense	5.376	25.900	-1.00	1712.64	7.62	Medium
19	4511350	T	A	*PLIN4*	0/1	.	missense	-1.002	0.016	0.78	9818.88	21.58	High
19	501743	T	C	*MADCAM1*	0/1	.	missense	-0.048	2.150	-0.65	394.94	4.18	Medium
19	4847740	T	C	*PLIN3*	0/1	.	missense	2.599	20.200	-0.91	119.10	2.37	Medium

19	48305586	G	A	*TPRX1*	0/1	.	missense	-0.648	0.095	-0.98	2120.83	8.48	Medium
19	46838160	A	G	*HIF3A*	0/1	.	splice acceptor	4.267	23.900	-	1871.98	7.96	Medium
20	31825582	G	A	*SPLUNC1*	0/1	.	missense	-2.838	0.001	0.43	18.06	0.63	Medium
20	46264232	C	A	*NCOA3*	0/1	.	missense	1.031	10.840	0.15	2649.42	9.66	Medium
20	33874475	C	T	*FAM83C*	0/1	.	missense	1.473	13.170	-0.22	2176.92	8.59	Medium
21	34927467	G	A	*SON‡*	0/1	.	missense	4.119	23.800	-1.00	1433.41	7.07	Medium
21	11038868	C	T	*LOC101926954*	0/1	.	missense	0.672	8.643		N/A	N/A	N/A
X	100749038	C	T	*ARMCX4*	0/1	.	missense	3.166	22.700	-0.98	1487.53	7.17	Medium
Other shared IBD variants													
1	228612890	G	A	*HIST3H3*	0/1	1.65E-05	missense	4.735	24.600	-0.97	156.55	2.73	Medium
1	145473471	C	T	*ANKRD34A*	0/1	8.24E-06	missense	4.286	24.000	-0.25	13.83	0.50	Medium
1	41236258	T	C	*NFYC*	0/1	8.24E-06	missense	1.251	12.020	-0.58	10.27	0.38	Medium
1	231487090	G	A	*SPRTN*	0/1	4.94E-05	missense	2.469	19.270	0.37	80.18	1.89	Medium
1	34554584	C	T	*CSMD2*	0/1	4.12E-05	missense	5.100	25.300	-1.00	4329.12	13.10	Medium
1	225340410	G	A	*D-H14*	0/1	0.007555	missense	2.898	21.800	-0.09	7454.49	18.57	High
1	45923405	C	T	*TESK2*	0/1	0.003691	missense	4.191	23.800	-0.97	210.44	3.13	Medium
1	70881670	C	T	*CTH*	0/1	0.006474	missense	6.745	32.000	-0.98	1259.00	6.69	Medium
1	155230432	G	A	*SCAMP3*	0/1	0.003986	missense	-0.328	0.573	-0.55	61.75	1.60	Medium
1	29010147	T	G	*GMEB1*	0/1	0.003303	missense	5.863	27.300	-1.00	29.05	0.93	Medium
1	216219841	G	T	*USH2A*	0/1	0.002257	missense	4.758	24.700	-0.99	6401.92	16.71	High
1	23417894	T	C	*LUZP1*	0/1	7.50E-04	missense	2.424	18.980	-0.89	889.13	5.81	Medium
1	17723586	C	T	*PADI6*	0/1	0.00177	stop gained	0.626	8.334	-	N/A	N/A	N/A
1	223934845	C	T	*CAPN2*	0/1	6.01E-04	missense	6.848	33.000	-1.00	3510.66	11.43	Medium
1	227920136	A	T	*JMJD4*	0/1	1.15E-04	missense	2.819	21.500	-0.98	1171.12	6.50	Medium
1	117658229	C	T	*TRIM45*	0/1	2.97E-04	missense	5.777	27.000	-1.00	3722.97	11.92	Medium
2	48132711	T	G	*FBXO11*	0/1	2.28E-04	missense	1.205	11.770	-0.58	113.87	2.33	Medium
2	26700556	C	T	*OTOF*	0/1	7.45E-05	missense	7.474	34.000	-0.99	7402.53	18.47	High
2	186661995	G	A	*FSIP2*	0/1	5.51E-05	missense	2.419	18.940	-1.00	5663.99	15.57	High
2	105713665	G	A	*MRPS9*	0/1	3.30E-05	missense	2.459	19.200	-0.73	109.26	2.27	Medium
2	48026101	A	G	*MSH6*	0/1	1.65E-05	missense	-1.229	0.006	0.18	1431.23	7.06	Medium
2	95818484	C	T	*ZNF514*	0/1	8.24E-06	missense	3.346	22.900	0.13	53.58	1.46	Medium

2	73479903	G	A	*CCT7*	0/1	5.79E-05	missense	1.739	14.630	-0.17	64.48	1.64	Medium
2	128784683	C	G	*SAP130*	0/1	1.34E-04	missense	4.952	25.000	-0.35	64.87	1.65	Medium
2	109432410	G	T	*CCDC138*	0/1	8.24E-06	missense	3.850	23.400	-0.44	2253.24	8.77	Medium
2	108994812	G	C	*SULT1C4*	0/1	8.24E-06	missense	0.260	5.295	-0.89	1571.30	7.34	Medium
2	29295186	C	T	*C2orf71*	0/1	0.009751	missense	0.432	6.890	-0.46	N/A	N/A	N/A
2	20130259	G	A	*WDR35*	0/1	0.00995	missense	5.074	25.300	-1.00	4168.69	12.81	Medium
2	84745113	C	T	*D-H6*	0/1	0.004521	missense	0.756	9.186	-	2649.62	9.66	Medium
2	33567971	C	T	*LTBP1*	0/1	0.00705	missense	7.357	34.000	-1.00	877.89	5.77	Medium
2	112725747	G	A	*MERTK*	0/1	.	missense	-0.832	0.036	0.09	1308.80	6.80	Medium
2	43903164	G	C	*LOC728819*	0/1	0.008093	missense	0.886	9.984	-	N/A	N/A	N/A
2	179605991	G	A	*TTN*	0/1	0.007001	missense	1.510	13.370	-	74772.87	42.91	High
2	169820801	C	T	*ABCB11*	0/1	0.002838	missense	5.183	25.500	-0.94	532.30	4.71	Medium
2	26407120	A	T	*GAREML*	0/1	0.007646	missense	5.307	25.800	-0.98	89.38	2.03	Medium
2	26541813	T	A	*GPR113*	0/1	0.007858	missense	0.717	8.939	-1.00	1162.28	6.49	Medium
2	27803089	T	C	*C2orf16*	0/1	0.005809	missense	1.096	11.200	-0.89	N/A	N/A	N/A
2	186656179	C	G	*FSIP2*	0/1	0.002866	missense	4.590	24.400	-1.00	5663.99	15.57	High
2	15534391	G	A	*NBAS*	0/1	0.003551	missense	6.533	31.000	-1.00	2659.86	9.69	Medium
2	185800661	G	A	*ZNF804A*	0/1	6.92E-04	missense	0.813	9.542	-0.79	13449.49	25.20	High
2	202356433	G	A	*ALS2CR11*	0/1	0.001598	missense	1.603	13.870	-0.22	653.81	5.13	Medium
2	65296693	G	C	*CEP68*	0/1	0.002471	missense	-1.492	0.003	-0.93	1991.19	8.22	Medium
2	103142757	G	A	*SLC9A4*	0/1	0.002899	missense	0.371	6.357	-0.84	448.62	4.40	Medium
2	67631910	G	T	*ETAA1*	0/1	4.37E-04	missense	3.212	22.700	-0.98	1000.99	6.10	Medium
3	129290532	C	T	*PLXND1*	0/1	4.86E-04	missense	3.931	23.500	-0.66	2739.77	9.87	Medium
3	78796044	C	T	*ROBO1*	0/1	8.28E-06	missense	7.135	34.000	-1.00	484.42	4.53	Medium
4	188924713	G	A	*ZFP42*	0/1	7.25E-04	missense	5.655	26.700	-0.89	35.38	1.06	Medium
4	189061031	G	A	*TRIML1*	0/1	6.59E-05	missense	3.029	22.300	-0.57	2068.55	8.38	Medium
4	187630099	C	T	*FAT1*	0/1	0.003641	missense	5.822	27.200	-1.00	12587.77	24.10	High
4	159789403	G	C	*FNIP2*	0/1	0.007212	missense	1.826	15.140	-0.73	731.03	5.37	Medium
5	90059270	C	A	*GPR98‡*	0/1	0.00245	missense	5.964	27.700	-1.00	32752.21	35.13	High
5	90001237	G	A	*GPR98‡*	0/1	0.001524	missense	6.971	33.000	-1.00	32752.21	35.13	High
5	80640844	G	A	*ACOT12*	0/1	0.01	missense	5.910	27.500	-0.25	944.27	5.95	Medium

5	178455114	A	G	*ZNF879*	0/1	0.004947	missense	-1.302	0.004	-	1265.83	6.71	Medium
5	156482296	C	G	*HAVCR1*	0/1	0.006928	missense	5.044	25.200	-1.00	2662.53	9.71	Medium
5	167489170	A	G	*TENM2*	0/1	0.005106	missense	1.830	15.160	-0.46	688.99	5.24	Medium
5	79024747	C	A	*CMYA5*	0/1	0.004065	missense	4.680	24.600	-1.00	21940.09	31.15	High
6	35420551	C	A	*FANCE*	0/1	2.57E-04	missense	4.863	24.900	-0.55	1627.90	7.46	Medium
6	32497961	T	G	*HLA-DRB5‡*	0/1	.	missense	-0.076	1.919	0.04	16958.18	27.73	High
6	32497962	T	C	*HLA-DRB5‡*	0/1	.	missense	1.281	12.170	-0.01	16958.18	27.73	High
6	90489962	G	C	*MDN1*	0/1	8.24E-05	missense	2.947	22.000	-	7714.21	18.90	High
6	152809550	T	C	*SYNE1*	0/1	2.47E-05	missense	-0.364	0.472	0.54	10210.55	22.01	High
6	139569043	T	C	*TXLNB*	0/1	0.001557	missense	1.929	15.770	-0.48	3565.93	11.55	Medium
6	32797726	C	T	*TAP2*	0/1	6.67E-04	missense	6.047	28.000	-0.39	2889.23	10.18	Medium
7	23299648	C	G	*GPNMB*	0/1	0.001697	missense	5.736	26.900	-0.99	437.62	4.36	Medium
7	4830203	C	T	*AP5Z1*	0/1	2.48E-04	missense	2.375	18.660	-0.74	1128.74	6.41	Medium
7	36466572	G	A	*ANLN*	0/1	4.94E-05	missense	1.707	14.450	-0.08	5386.34	15.14	High
7	31862756	T	C	*PDE1C*	0/1	0.008072	missense	0.950	10.370	-0.80	247.32	3.39	Medium
7	4014039	C	T	*SDK1*	0/1	0.008986	missense	2.438	19.070	-0.78	1933.80	8.09	Medium
7	100675940	A	G	*MUC17*	0/1	0.003657	missense	1.013	10.730	-	4366.27	13.19	Medium
7	100685634	C	T	*MUC17*	0/1	0.001721	missense	0.154	4.190	-	4366.27	13.19	Medium
7	100091306	C	T	*NYAP1*	0/1	0.001368	missense	3.617	23.200	-0.98	99.94	2.17	Medium
8	16884980	G	A	*MICU3*	0/1	1.72E-05	stop gained	10.044	36.000	-	1124.89	6.40	Medium
8	92213022	T	A	*LRRC69*	0/1	0.009095	splice donor	3.646	23.200	-	72.62	1.78	Medium
8	11659451	G	C	*RP11-297N6.4*	0/1	0.001957	missense	2.411	18.890	-1.00	1422.06	7.04	Medium
8	145107390	C	T	*OPLAH*	0/1	0.006353	missense	6.131	28.400	-0.95	2315.29	8.91	Medium
8	22049596	G	A	*BMP1*	0/1	0.003706	missense	7.046	33.000	-0.46	714.38	5.31	Medium
8	22168677	C	T	*PIWIL2*	0/1	0.005593	missense	5.276	25.700	-0.90	286.92	3.63	Medium
8	38162945	G	A	*WHSC1L1*	0/1	0.007059	missense	5.869	27.400	-0.10	249.03	3.40	Medium
8	145140564	G	A	*GPAA1*	0/1	0.005244	missense	1.809	15.040	-0.93	269.44	3.52	Medium
8	144658630	G	A	*-PRT1*	0/1	0.004937	missense	6.987	33.000	-1.00	589.28	4.92	Medium
8	30702739	G	C	*TEX15*	0/1	0.004209	missense	2.599	20.200	-0.62	4622.32	13.67	Medium
8	145736819	G	A	*RECQL4*	0/1	5.54E-04	missense	3.879	23.500	-1.00	7865.47	19.17	High
8	21981305	T	C	*HR*	0/1	3.81E-04	missense	2.578	19.960	-0.97	2359.44	9.01	Medium

8	145730072	G	A	*GPT*	0/1	1.40E-04	missense	7.132	34.000	-1.00	2186.35	8.61	Medium
8	67771662	T	G	*SGK3*	0/1	8.48E-04	missense	6.640	32.000	-0.95	N/A	N/A	N/A
9	91606398	C	G	*C9orf47*	0/1	0.006337	missense	2.345	18.460	-	N/A	N/A	N/A
9	133768966	C	T	*QRFP*	0/1	0.006606	missense	2.074	16.680	-0.90	120.58	2.39	Medium
9	132374704	C	A	*C9orf50*	0/1	0.009111	missense	2.100	16.850	-0.29	N/A	N/A	N/A
9	99522502	C	T	*ZNF510*	0/1	0.001796	missense	-0.654	0.092	0.06	1487.98	7.18	Medium
9	86616901	A	G	*RMI1*	0/1	0.002726	missense	-2.160	0.001	0.35	98.05	2.14	Medium
9	114246471	C	G	*KIAA0368*	0/1	0.008615	missense	0.660	8.565	-1.00	4460.55	13.39	Medium
9	98638328	C	A	*ERCC6L2*	0/1	0.005651	missense	0.735	9.051	-0.88	3928.78	12.39	Medium
9	13223592	G	C	*MPDZ*	0/1	0.00173	missense	4.180	23.800	-0.99	1523.84	7.25	Medium
10	46965018	C	G	*SYT15*	0/1	.	missense	0.444	6.987	-0.58	417.37	4.28	Medium
10	1279718	C	G	*ADARB2*	0/1	1.89E-04	missense	-1.392	0.003	0.08	584.20	4.90	Medium
10	113926224	A	G	*GPAM*	0/1	0.01	missense	3.764	23.300	-0.41	242.46	3.36	Medium
10	101557063	G	A	*ABCC2*	0/1	0.005593	missense	4.063	23.700	-1.00	1270.68	6.72	Medium
10	112269908	C	G	*DUSP5*	0/1	0.01	missense	4.070	23.700	-0.32	292.05	3.66	Medium
10	129904286	G	A	*MKI67*	0/1	0.004909	missense	0.995	10.630	-0.94	13447.48	25.13	High
10	101715249	T	C	*DNMBP*	0/1	0.004711	missense	-0.675	0.082	-0.70	1197.05	6.56	Medium
10	104359297	G	T	*SUFU*	0/1	0.006046	missense	0.999	10.660	-0.05	42.64	1.24	Medium
10	76910529	T	A	*SAMD8*	0/1	0.004283	missense	0.721	8.964	0.18	31.66	1.00	Medium
10	102255216	G	C	*SEC31B*	0/1	0.004332	missense	1.698	14.400	-0.70	8025.98	19.31	High
10	120931928	C	A	*PRDX3*	0/1	0.005181	missense	8.318	35.000	-1.00	110.77	2.29	Medium
10	70179634	G	T	*D-2*	0/1	0.004454	missense	2.576	19.960	-0.15	1134.53	6.43	Medium
10	111890151	C	T	*ADD3*	0/1	0.001021	missense	2.922	21.900	-0.73	379.33	4.11	Medium
10	123970722	T	C	*TACC2*	0/1	0.002899	missense	2.904	21.900	-0.59	4233.02	12.94	Medium
10	79745718	C	T	*POLR3A*	0/1	2.47E-05	missense	6.350	29.400	-0.93	227.15	3.26	Medium
11	1911355	C	T	*C11orf89*	0/1	9.56E-05	missense	0.647	8.479	-0.34	N/A	N/A	N/A
11	120827611	G	T	*GRIK4*	0/1	8.24E-06	missense	6.587	31.000	-1.00	476.06	4.51	Medium
11	5602438	T	A	*OR52B6*	0/1	.	missense	0.301	5.700	0.06	210.58	3.13	Medium
11	125830970	A	T	*CDON*	0/1	.	missense	-0.119	1.592	0.90	2210.62	8.65	Medium
11	111636995	C	T	*PPP2R1B*	0/1	1.57E-04	missense	8.098	35.000	-1.00	326.85	3.84	Medium
11	12315175	G	A	*MICALCL*	0/1	0.0073	missense	0.114	3.754	-0.24	915.20	5.88	Medium

11	118307316	C	G	*KMT2A*	0/1	0.008242	missense	2.432	19.030	-0.83	416.02	4.27	Medium
11	108032676	G	A	*NPAT*	0/1	0.00466	missense	2.868	21.700	-0.97	1731.00	7.68	Medium
11	108277861	C	T	*C11orf65*	0/1	0.00463	missense	2.868	21.700	-0.94	N/A	N/A	N/A
11	6191528	T	C	*OR52B2*	0/1	0.006698	missense	0.753	9.170	-0.94	579.94	4.88	Medium
11	111784401	G	A	*HSPB2-C11orf52*	0/1	0.006721	missense & NMD transcript	6.755	32.000	-1.00	N/A	N/A	N/A
11	7960232	G	A	*OR10A3*	0/1	0.003435	missense	1.939	15.830	-0.98	136.22	2.54	Medium
11	114310345	C	T	*REXO2*	0/1	0.003002	missense	3.140	22.600	-1.00	22.41	0.75	Medium
11	6150210	T	C	*OR56B3P*	0/1	8.98E-04	missense	1.483	13.220	-0.07	80.88	1.90	Medium
11	1605919	G	T	*KRTAP5-1*	0/1	.	stop gained	6.943	33.000	-	70.10	1.74	Medium
11	126075477	C	G	*RPUSD4*	0/1	2.72E-04	missense	1.584	13.760	-0.94	2854.48	10.11	Medium
12	5155046	G	A	*KC-5*	0/1	0.005354	missense	0.664	8.590	-0.31	249.25	3.40	Medium
12	109577344	A	G	*ACACB*	0/1	0.002051	missense	-0.057	2.073	0.62	3979.36	12.48	Medium
12	128899991	A	G	*TMEM132C*	0/1	1.07E-04	missense	0.334	6.016	0.44	3428.15	11.24	Medium
13	53261947	G	T	*SUGT1*	0/1	1.65E-05	missense	5.405	26.000	-0.43	22.99	0.76	Medium
13	107823070	T	A	*FAM155A*	0/1	0.007133	missense	-1.048	0.013	-0.54	761.08	5.47	Medium
13	25842016	C	G	*MTMR6*	0/1	0.007965	missense	0.771	9.285	0.15	4034.99	12.59	Medium
13	60485919	A	G	*DIAPH3*	0/1	0.003684	missense	3.879	23.500	0.42	1035.20	6.17	Medium
13	32945172	A	C	*BRCA2*	0/1	8.81E-04	missense	3.832	23.400	-0.72	775.59	5.51	Medium
14	51348345	G	T	*ABHD12B*	0/1	0.008549	splice donor	3.877	23.500	-	4353.14	13.15	Medium
14	24724656	T	C	*TGM1*	0/1	0.005667	missense	5.819	27.200	-0.60	277.28	3.56	Medium
14	77697979	C	G	*TMEM63C*	0/1	9.04E-04	missense	2.027	16.380	-0.33	68.68	1.71	Medium
15	56387606	C	G	*RFX7*	0/1	0.007358	missense	1.287	12.200	0.01	372.49	4.07	Medium
15	59806496	G	T	*FAM81A*	0/1	0.008449	missense	5.021	25.200	-0.92	61.36	1.60	Medium
15	34649336	G	A	*NUTM1*	0/1	0.002323	missense	4.126	23.800	-0.85	2356.46	9.00	Medium
15	89382008	C	A	*ACAN*	0/1	4.30E-04	missense	2.695	20.800	-0.65	15528.89	26.89	High
16	89017617	G	C	*RP11-830F9.6‡*	0/1	2.75E-04	missense	-0.344	0.525	-	1805.30	7.84	Medium
16	89017620	C	G	*RP11-830F9.6‡*	0/1	1.70E-04	missense	0.189	4.566	-	1805.30	7.84	Medium
16	4383414	A	T	*GLIS2*	0/1	5.69E-04	missense	5.986	27.800	-0.99	614.21	5.01	Medium
16	78062044	G	C	*CLEC3A*	0/1	1.32E-04	missense	5.063	25.200	-0.06	627.36	5.05	Medium
16	77325185	C	T	*ADAMTS18*	0/1	3.30E-05	stop gained	14.112	44.000	-	12292.97	23.78	High

16	46652227	G	C	*SHCBP1*	0/1	0.008245	missense	0.736	9.060	-0.54	50.36	1.39	Medium
16	31090269	C	T	*ZNF646*	0/1	0.009991	missense	1.969	16.020	-0.74	5474.89	15.25	High
16	20442346	A	C	*ACSM5*	0/1	0.004851	missense	1.107	11.260	-0.90	7462.84	18.58	High
16	22149688	T	C	*VWA3A*	0/1	0.005957	missense	4.098	23.700	-0.53	967.81	6.02	Medium
16	72107691	G	A	*HPR*	0/1	0.007917	missense	-0.012	2.472	-	536.91	4.72	Medium
16	53321892	A	G	*CHD9*	0/1	0.002724	missense	2.878	21.800	-0.93	1762.92	7.75	Medium
16	11272482	G	A	*CLEC16A*	0/1	0.002094	missense	-0.187	1.166	-0.45	250.55	3.41	Medium
16	4745145	A	G	*NUDT16L1‡*	0/1	0.001005	missense	1.695	14.380	-0.90	12.74	0.46	Medium
16	4745146	A	C	*NUDT16L1‡*	0/1	9.80E-04	missense	2.432	19.030	-0.93	12.74	0.46	Medium
17	43319274	C	T	*FMNL1*	0/1	4.17E-04	missense	2.448	19.130	-0.96	3176.17	10.74	Medium
17	19646144	C	T	*ALDH3A1*	0/1	1.74E-05	missense	0.081	3.405	-	3022.03	10.44	Medium
17	37604062	G	T	*MED1*	0/1	8.24E-06	missense	7.217	34.000	-1.00	103.17	2.19	Medium
17	78211376	C	T	*SLC26A11*	0/1	2.06E-04	missense	5.894	27.400	-0.99	810.57	5.60	Medium
17	59793412	G	A	*BRIP1*	0/1	1.48E-04	stop gained	12.661	39.000	-	155.98	2.73	Medium
17	61278229	T	G	*TANC2*	0/1	0.009666	missense	0.009	2.674	-0.45	191.80	3.00	Medium
17	43906973	G	A	*CRHR1*	0/1	0.006364	missense	0.971	10.500	-0.93	63.73	1.64	Medium
17	17070194	G	C	*MPRIP*	0/1	0.007299	missense	-0.527	0.189	-0.63	N/A	N/A	N/A
17	36959002	G	A	*CWC25*	0/1	0.009475	missense	5.065	25.200	-0.64	76.65	1.84	Medium
17	74154496	G	A	*RNF157*	0/1	0.007388	missense	0.824	9.609	-0.40	274.87	3.55	Medium
17	16527530	A	G	*ZNF624*	0/1	0.007965	missense	-0.024	2.366	-0.96	2684.49	9.74	Medium
17	37871547	C	A	*ERBB2*	0/1	0.003451	missense	1.871	15.410	-0.15	5901.31	15.95	High
17	45886471	G	A	*OSBPL7*	0/1	7.17E-04	missense	1.909	15.640	-	151.61	2.69	Medium
17	72368467	C	T	*GPR142*	0/1	0.003328	missense	6.205	28.700	-1.00	583.16	4.90	Medium
17	48618931	C	G	*EPN3*	0/1	1.81E-04	missense	0.592	8.099	-0.84	3548.81	11.51	Medium
17	72791746	G	A	*TMEM104*	0/1	6.18E-04	missense	7.552	34.000	-0.98	208.94	3.12	Medium
18	12262897	C	T	*CIDEA*	0/1	0.002767	missense	7.874	35.000	-1.00	171.63	2.86	Medium
18	55992249	T	A	*NEDD4L*	0/1	5.70E-04	missense	2.036	16.440	-0.83	42.02	1.22	Medium
19	40541778	C	T	*ZNF780B*	0/1	4.04E-04	missense	5.281	25.700	-0.97	467.55	4.48	Medium
19	18330082	A	G	*PDE4C*	0/1	2.47E-05	missense	2.978	22.200	-0.99	2015.83	8.27	Medium
19	39663757	G	A	*PAK4*	0/1	0.008161	missense	-0.831	0.037	-0.32	120.47	2.39	Medium
19	17108127	C	T	*CPAMD8*	0/1	0.007214	missense	2.867	21.700	-0.95	4982.63	14.40	High

19	17945696	C	T	*JAK3*	0/1	0.008632	missense	1.998	16.200	-0.12	301.14	3.70	Medium
19	44153248	T	C	*PLAUR*	0/1	0.003171	missense	3.286	22.800	-0.37	2238.42	8.73	Medium
19	9324678	G	T	*OR7D4*	0/1	0.003484	missense	2.173	17.330	-0.07	1688.92	7.58	Medium
19	13409407	C	T	*CAC-1A*	0/1	0.002	missense	2.089	16.780	-0.70	4523.78	13.51	Medium
19	49360698	C	T	*PLEKHA4*	0/1	0.001412	missense	3.339	22.900	-0.82	3929.35	12.39	Medium
19	58437573	C	T	*ZNF418*	0/1	9.64E-04	missense	0.334	6.018	-0.66	147.27	2.64	Medium
19	55451438	A	C	*NLRP7*	0/1	3.95E-04	missense	3.486	23.100	-1.00	142.92	2.61	Medium
20	47852689	G	A	*DDX27*	0/1	5.77E-05	missense & splice region	6.871	33.000	-0.97	4157.56	12.80	Medium
20	55750060	T	C	*BMP7*	0/1	0.00537	missense	-1.131	0.009	-0.03	29.85	0.95	Medium
20	49576664	T	G	*MOCS3*	0/1	0.00626	missense	-2.193	0.001	0.10	58.75	1.55	Medium
20	2321138	C	T	*TGM3*	0/1	0.002594	missense	7.364	34.000	-0.78	1529.88	7.26	Medium
20	44049008	C	T	*PIGT*	0/1	5.44E-04	missense	6.077	28.100	-0.50	479.41	4.52	Medium
21	27071018	C	A	*JAM2*	0/1	2.E-04	missense	5.998	27.800	-1.00	364.51	4.04	Medium
21	45649714	C	T	*ICOSLG*	0/1	7.E-03	missense	-0.354	0.497	-	2097.37	8.44	Medium
21	34923617	A	G	*SON‡*	0/1	4.E-03	missense	2.119	16.980	-1.00	1433.41	7.07	Medium
22	24761456	G	A	*SPECC1L*	0/1	8.E-06	missense	5.358	25.900	-0.88	294.44	3.67	Medium
22	19127540	G	C	*DGCR14*	0/1	7.E-05	missense & splice region	1.132	11.390	-	1245.24	6.67	Medium
22	26565686	C	A	*SEZ6L*	0/1	3.E-03	missense	2.021	16.340	0.54	1299.61	6.78	Medium
22	45258284	G	C	*PRR5-ARHGAP8*	0/1	3.E-03	missense	2.133	17.070	-0.93	986.92	6.06	Medium
22	30518166	G	A	*HORMAD2*	0/1	2.E-03	missense	4.144	23.800	0.12	116.45	2.35	Medium
22	20024596	C	G	*TANGO2*	0/1	6.E-03	stop gained	0.856	9.803	-	155.69	2.73	Medium
X	29959846	A	G	*IL1RAPL1*	0/1	4.E-04	missense			-1.00	15.07	0.54	Medium

‡ Shared compound variants
0/1 Heterozygous variant
1/1 Homozygous variant

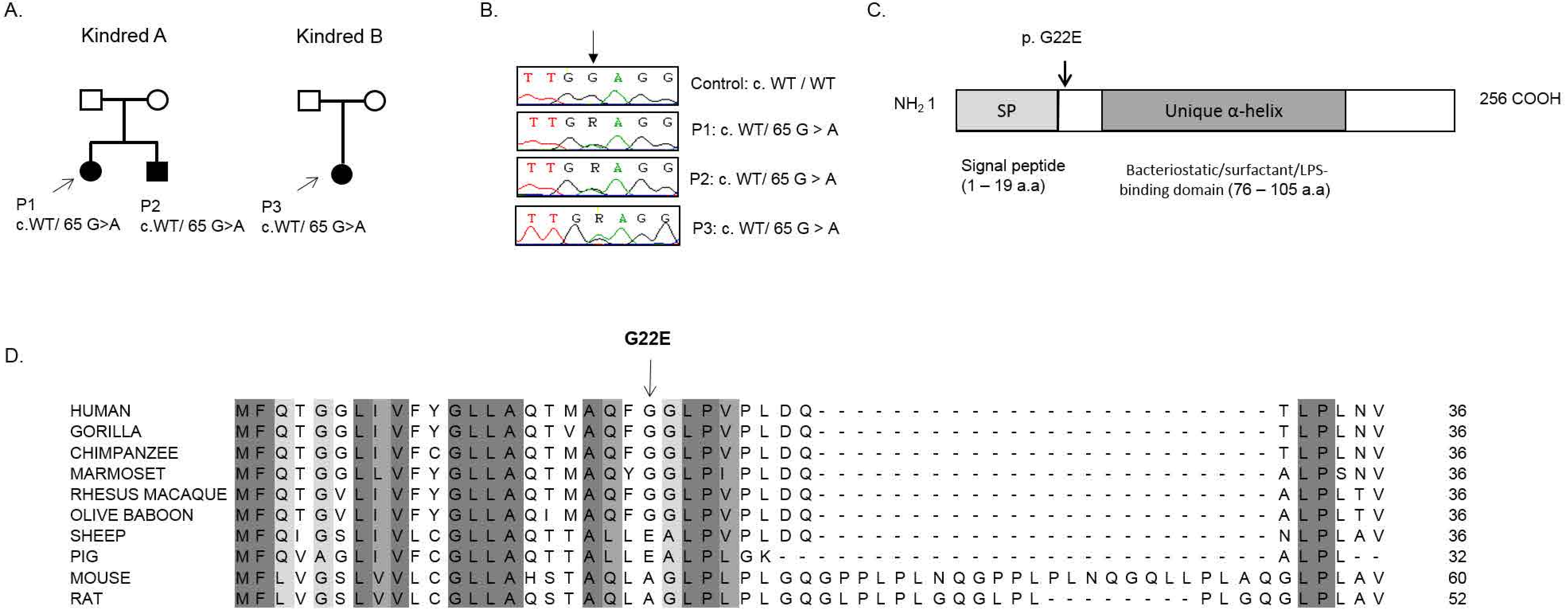

A.
Kindred A
Kindred B
P1
c.WT/ 65 G>A
P2
c.WT/ 65 G>A
P3
c.WT/ 65 G>A
B.
T T G G A G G
Control: c. WT / WT
T T G R A G G
P1: c. WT/ 65 G > A
T T G R A G G
P2: c. WT/ 65 G > A
T T G R A G G
P3: c. WT/ 65 G > A
C.
p. G22E
NH_2 1
SP
Unique α-helix
256 COOH
Signal peptide
(1 – 19 a.a)
Bacteriostatic/surfactant/LPS-
binding domain (76 – 105 a.a)
D.
G22E
HUMAN M F Q T G G L I V F Y G L L A Q T M A Q F G G L P V P L D Q - - - T L P L N V 36
GORILLA M F Q T G G L I V F Y G L L A Q T V A Q F G G L P V P L D Q - - - T L P L N V 36
CHIMPANZEE M F Q T G G L I V F C G L L A Q T M A Q F G G L P V P L D Q - - - T L P L N V 36
MARMOSET M F Q T G G L L V F Y G L L A Q T M A Q Y G G L P I P L D Q - - - A L P S N V 36
RHESUS MACAQUE M F Q T G V L I V F Y G L L A Q T M A Q F G G L P V P L D Q - - - A L P L T V 36
OLIVE BABOON M F Q T G V L I V F Y G L L A Q I M A Q F G G L P V P L D Q - - - A L P L T V 36
SHEEP M F Q I G S L I V L C G L L A Q T T A L L E A L P V P L D Q - - - N L P L A V 36
PIG M F Q V A G L I V F C G L L A Q T T A L L E A L P L G K - - - A L P L - - 32
MOUSE M F L V G S L V V L C G L L A H S T A Q L A G L P L P L G Q G P P L P L N Q G P P L P L N Q G Q L L P L A Q G L P L A V 60
RAT M F L V G S L V V L C G L L A Q S T A Q L A G L P L P L G Q G L P L P L G Q G L P L - - - P L G Q G L P L A V 52
